# Transcriptome analysis of mulberry (*Morus alba* L.) leaves to identify differentially expressed genes associated with post-harvest shelf-life elongation

**DOI:** 10.1038/s41598-022-21828-7

**Published:** 2022-10-28

**Authors:** Dipayan Das, Subires Bhattacharyya, Monidipa Bhattacharyya, Puja Sashankar, Arindam Ghosh, Palash Mandal

**Affiliations:** 1 Plant Physiology and Pharmacognosy Research Laboratory, Department of Botany, University of North Bengal, Siliguri, West Bengal 734013 India; 2 Nanobiology and Phytotherapy Laboratory, Department of Botany, University of North Bengal, Siliguri, West Bengal 734013 India; 3 Department of Botany, Government General Degree College, Gorubathan, Fagu, Kalimpong, 735231 India; 4 Centre of Bioinformatics, Institute of Interdisciplinary Studies, University of Allahabad, Prayagraj, 211002 India; 5 Institute of Biomedicine, University of Eastern Finland, 70210 Kuopio, Finland

**Keywords:** Biochemistry, Physiology, Plant sciences

## Abstract

Present study deals with molecular expression patterns responsible for post-harvest shelf-life extension of mulberry leaves. Quantitative profiling showed retention of primary metabolite and accumulation of stress markers in NS7 and CO7 respectively. The leaf mRNA profiles was sequenced using the Illumina platform to identify DEGs. A total of 3413 DEGs were identified between the treatments. Annotation with Arabidopsis database has identified 1022 DEGs unigenes. STRING generated protein–protein interaction, identified 1013 DEGs nodes with *p* < 1.0e−16. KEGG classifier has identified genes and their participating biological processes. MCODE and BiNGO detected sub-networking and ontological enrichment, respectively at *p* ≤ 0.05. Genes associated with chloroplast architecture, photosynthesis, detoxifying ROS and RCS, and innate-immune response were significantly up-regulated, responsible for extending shelf-life in NS7. Loss of storage sucrose, enhanced activity of senescence-related hormones, accumulation of xenobiotics, and development of osmotic stress inside tissue system was the probable reason for tissue deterioration in CO7. qPCR validation of DEGs was in good agreement with RNA sequencing results, indicating the reliability of the sequencing platform. Present outcome provides a molecular insight regarding involvement of genes in self-life extension, which might help the sericulture industry to overcome their pre-existing problems related to landless farmers and larval feeding during monsoon.

## Introduction

A cumulative survey of different textile-based industries ranks silk as the most elegant textile in the world and is recognized as the "Queen of Textiles"^1,2^. Sericulture practice continues to remain promising rural activity in silk-rearing states as it generates maximum employment with minimal investment and minimum gestation period, and shows quick turnover potential^3^. Recently the number of participating workers in sericulture practice gradually decreased due to rapid urbanization^4^. Landless sericulture workers either work in the gardens of other seri-farmers or, if willing to run their rearing practice, they have to purchase leaves from others' gardens regularly. The cost of transporting plucked leaves decreases the profit ratio of the overall rearing process. As a result, young seri-farmers leave their traditional practice by moving to urban areas for employment. A decrease in productivity (cocoon formation) due to increased larval mortality during the rainy season by feeding wet leaves was another reason for decreasing interest and urban mobilization by seri-farmers.

Post-harvest leaf preservation may help landless farmers as they can carry out their rearing practices by collecting leaves occasionally. Besides this, feeding fresh preserved leaves during the rainy season may prevent depletion of productivity as it will ensure larval survival without developing the disease.

Delimitations of post-harvest preservation are leaf wilting, chloroplast discoloration indicating senescence, high respiration rate, decay of tissue, and microbial growth blocking conducting pathway^5,6^. Merzlyak and Hendry^7^ reported that an increase in ROS and free radical accumulation due to microbial proliferation promotes macromolecules' rapid degradation, accelerating senescence. Das and Roychoudhury^8^ stated that ROS accumulation above threshold level causes damage at cellular level which is apparent by degradation of pigment, carbohydrates, proteins, lipids, and even nucleic acid. The breakdown of photosynthetic pigments primarily categorizes senescence, resulting in a gradual decrease in photosynthetic rate and photosynthesis to respiration ratio^9^. It was reported that senescence causes isolation of chloroplast and generation of O_2_^·−^ through reduction of cellular O_2_ by means of photosynthetic electron transport chain^10^. Generated O_2_^·−^ causes cell and organelle damage^11,12^ by promoting the process of lipid peroxidation^7,13^.

At the onset of stress, the plant adopts several senescence retardation cellular mechanisms. Our earlier studies in this aspect have determined phytosynthesized silver nanoparticles as a preservative solution in prolonging the shelf life of mulberry leaves by seven days. Observations of our studies reveal that photosynthesized silver nanoparticles using mulberry leaf extract prevent the formation of xylem blockage by inhibiting microbial proliferation^14^, thereby maintaining conducting column continuity. Nanoparticles as preservative retains uniformity of photosynthetic apparatus and primary metabolite content. It nullifies generated reactive oxygen species (ROS) by activating enzymatic and non-enzymatic antioxidant-based defensive activities^13^.

The present study attempted to identify probable defensive lines in the post-harvest shelf-life extension of nanosilver-mediated preserved mulberry leaves through gene expression analysis. Next-generation sequencing (NGS) platform provides an opportunity to identify probable genetic mechanisms involved in any biological, cellular, or molecular processes^15^. NGS platform also allows the study of transcriptome sequencing of model and non-model organisms^16^. In biological studies, NGS-generated data was considered an ideal tool for gene expression analysis, discovering novel genes, and developing molecular markers^17^. Transcriptome-mediated gene expression analysis has been successfully implemented in several economically important plants, including rice^18^, wheat^19^, maize^20^, oat^21^, jackfruit^22^, spinach^23^, pear^24^, raspberry^25^, and many others.

In this study, comparative transcriptome profile-based characterization of 7 days nanosilver and distilled water preserved mulberry leaves was conducted using a high-throughput Illumina platform for fulfilling three objectives. Firstly, to identify differentially expressed up-regulated and down-regulated genes; secondly, to determine the functional identity of differentially expressed genes; and finally, to identify probable defensive lines responsible for extending post-harvest shelf-life of mulberry leaves preserved in nanosilver solution.

## Results

### Response of mulberry leaves in post-harvest preservative solution

**Figure 1:**
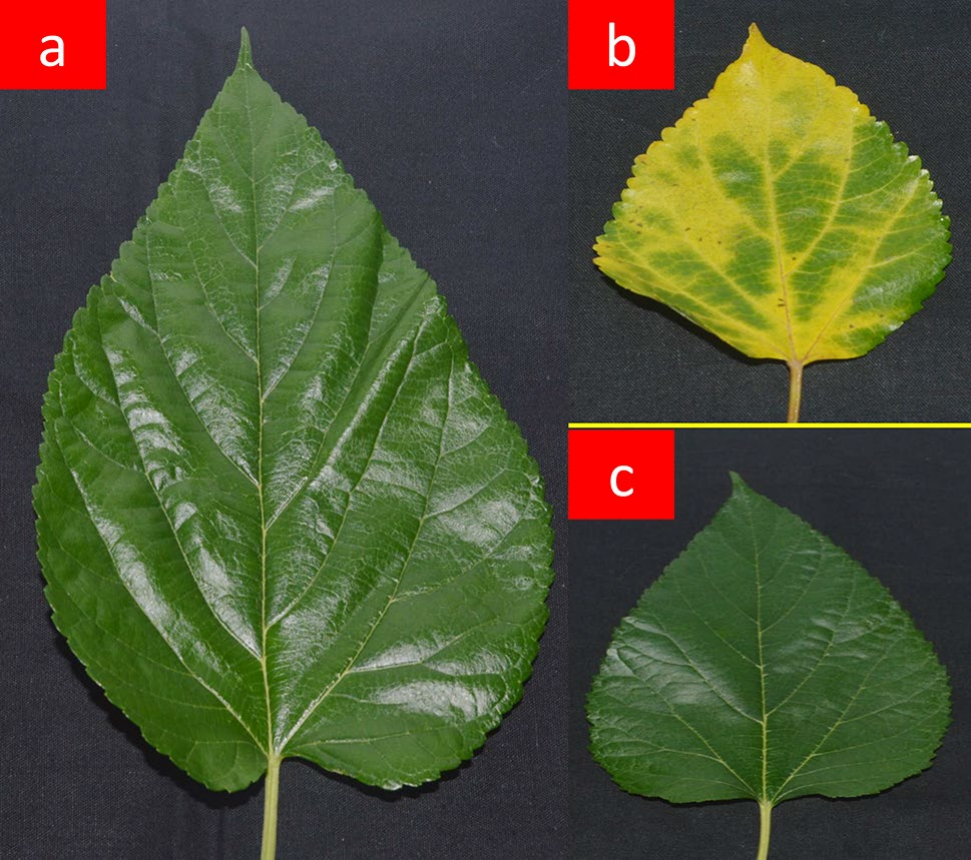
Physical texture of mulberry leaves at the end of preservation period in comparison with fresh leaves. (**A**) Fresh leaf, (**B**) 7 day preserved leaf in distilled water and (**C**) 7 day preserved leaf in nanosilver solution.

Nanosilver solution showed greater preservative potentiality than distilled water in preserving mulberry leaves at the post-harvest stage. In comparison to the initial day (FR0), leaves preserved for seven days in nanosilver solution (NS7) displayed greater retention of fresh texture than distilled water preserved (CO7) leaves (Fig. 1). The presence of prominent yellowish patches in CO7 displayed an indicative signal regarding activation of the senescence processes.

**Figure 2:**
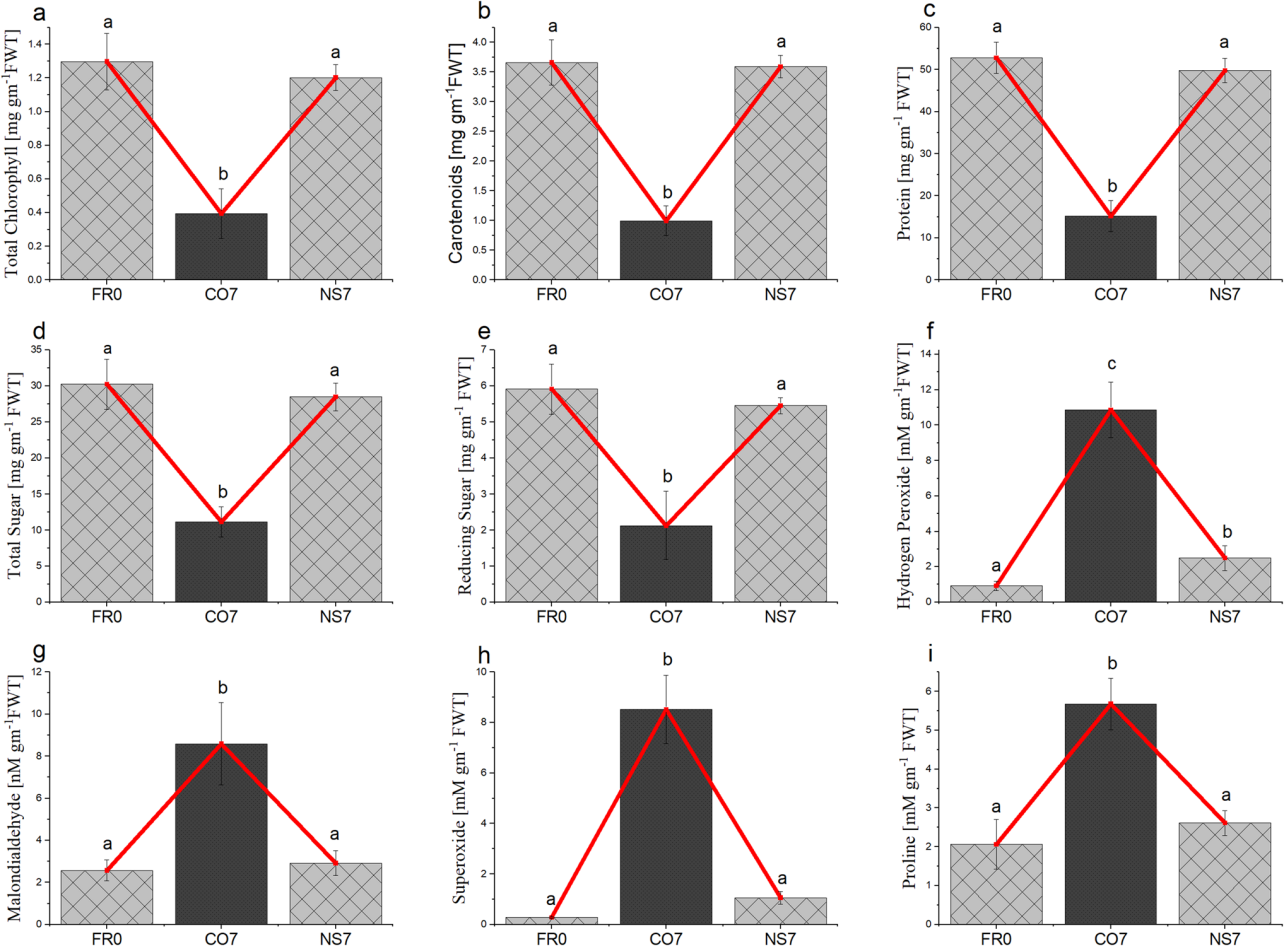
Comparative evaluation of (**A**) total chlorophyll, (**B**) carotenoids, (**C**) total protein, (**D**, **E**) total and reducing sugar, (**F**) hydrogen peroxide, (**G**) Malondialdehyde, (**H**) superoxide and (**I**) proline content between 7 day preserved leaves in distilled water (CO7) and nanosilver solution (NS7) with respect to fresh leaves (FR0). The results were expressed as Mean ± SDEV, n = 3. Values with different letters (a, b, c, etc.) differ significantly (*p* ≤ 0.05) by Duncan’s Multiple Range Test (DMRT).

The content of phyto-metabolites viz. total chlorophyll, carotenoids, total protein, total sugar, and reducing sugar in NS7 remains almost similar to that of initial day. In contrast, a greater decrease in the content was recorded for CO7 (Fig. 2). Accumulation of stress indicators viz. hydrogen peroxide, MDA, superoxide, and proline were recorded to be significantly greater in CO7 than NS7. With respect to the initial day, the content of total chlorophyll, carotenoids, total protein, total sugar, and reducing sugar in CO7 decreased by approximately 69, 72, 71, 63, and 64%; while in NS7 by around 7, 1, 5, 5, and 7% respectively. Principle Component Analysis positioned both NS7 and CO7 within the first component (PC1) (Supplementary Fig. 1). CO7 occupied the positive axis of PC1 with an axis point at + 4.2223. In contrast, NS7 positioned itself in the negative axis along with FR0 having axis points at -1.7798 and -2.4425 respectively.

### Illumina paired-end sequencing and de novo transcriptome assembly

**Figure 3:**
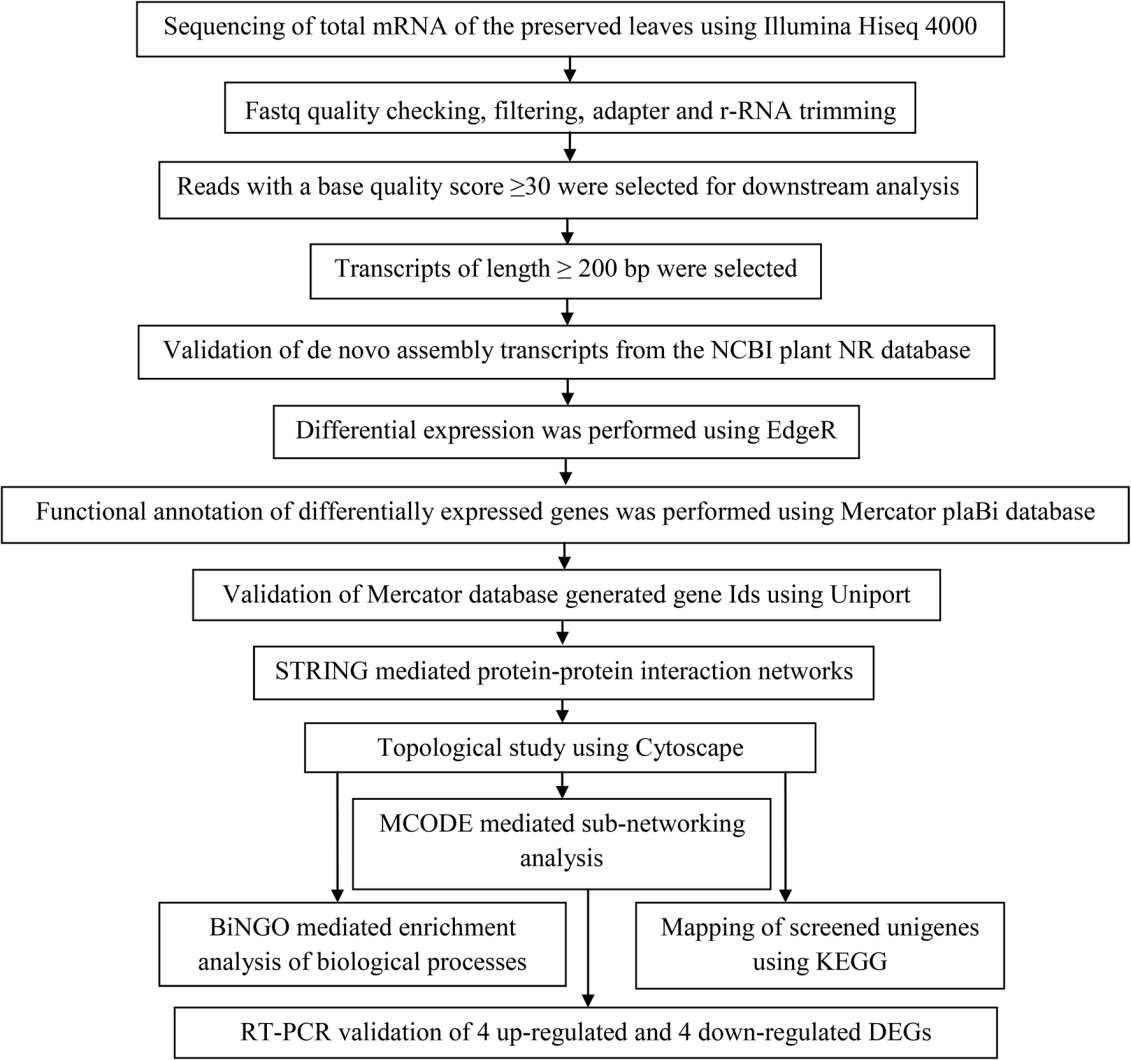
Flowchart describing the steps of data procession and subsequent analysis of differentially expressed genes.

Total mRNA from the preserved leaves of S1 mulberry cultivars, NS7 and CO7, were sequenced using Hiseq 4000. Illumina sequencing of mulberry transcriptome yielded a raw read of 96411054 and 94997804 for NS7 and CO7, respectively, totaling around 191 million (Table 1). The average base quality was above Q30 (error-probability ≥ 0.001) for 93.81% of bases. The %GC content of the reads within the test sample followed a regular distribution pattern. The average Q30 percentage for NS7 and CO7 was 95.63, and 96.43%, and GC percentages were 48.19 and 47.76%, respectively. Prior to transcriptome assembly, the raw reads were trimmed to remove any adapter sequences (AdapterRemoval 2. 2.0), and the reads with an average quality score of less than 20 were filtered out. The rRNA sequences were also removed using the Silva database (Table 2). On average, nearly 71% of processed high-quality reads with a base quality score ≥ 30 were obtained that were used for downstream analysis. The clean reads were submitted to the National Centre for Biotechnology Information (NCBI) which can be accessed from the sequenced read archives (SRA) under accession numbers SRR9665629 and SRR9665368 for NS7 and CO7, respectively. The SRA was registered under the same bio-project and bio-sample accession numbers PRJNA553319 and SAMN12234591, respectively. The subsequent steps of data procession and analysis of differentially expressed genes are represented schematically in Fig. 3.

**Table 1:** Sequencing statistics of mulberry transcriptomes.

Sample	Mean read quality (Phred score)	Number of raw reads	% GC	% Q < 10	% Q 10–20	% Q 20–30	% Q > 30	Number of bases (MB)	Mean read length (bp)
NS 7	39.24	96411054	48.19	0	1.6	2.79	95.63	9641.1	100
CO7	39.48	94997804	47.76	0	1.26	2.29	96.43	9499.78	100

**Table 2:** Summary of paired-end RNA sequences of preserved mulberry leaves.

Sample	Raw reads	% Raw read	Processed reads (adapter trimmed and quality trimmed)	% Processed read	Reads after rRNA removal	% Read after rRNA removal
NS7	96411054	100	96403161	99.992	42683018	44.276
CO7	94997804	100	94951582	99.951	94494544	99.519

The cleaned reads were used for transcriptome assembly using Trinity (Trinity2.8.2). Transcripts of length ≥ 200 bp were selected and considered for downstream analysis. The Trinity program generated 157982 assembled transcripts (isoforms) and 81952 unigenes with the longest transcript length (bp) of 24890 and 79047, respectively (Supplementary Table 1). The quality of assembled transcriptome was supported by N50 values of 2054 bp and 1374 bp for isoforms and unigenes, respectively (Supplementary Table 2). Based on the transcript length, 35.60% (29175) unigenes were < 300 bp, 53.22% (43617) were between 300 and 2000 bp, 9.08% (7442) were between 2000 and 5000 bp, while 2.09% (1718) unigenes were > 5000 bp (Supplementary Fig. 2). The average GC content was around 40% (Supplementary Fig. 3). Based on the GC range, the transcripts were categorized this way: 5471 transcript unigenes have a GC range < 30%; 69512, and 6873 unigenes have a GC range between 30–50% and 50–70%, respectively; while nine unigenes have a GC range > 70%.

### Annotation of unigenes

Assembled unigenes were annotated and validated from the NCBI plant NR database using blastx (Supplementary Dataset S1). Blastx found hits in the NR database detected 55.24% (45273) unigenes out of the total number of 81952 unigenes (E-value ≤ 1.42E–5). Among the annotated unigenes, 9795 and 6931 hypothetical and uncharacterized proteins were detected, respectively. In addition, the plant metabolic network (PMN) has detected 46440 unigenes, accounting for 56.66% of the total (E-value ≤ 1.93E−5) (Supplementary Dataset S2).

**Figure 4:**
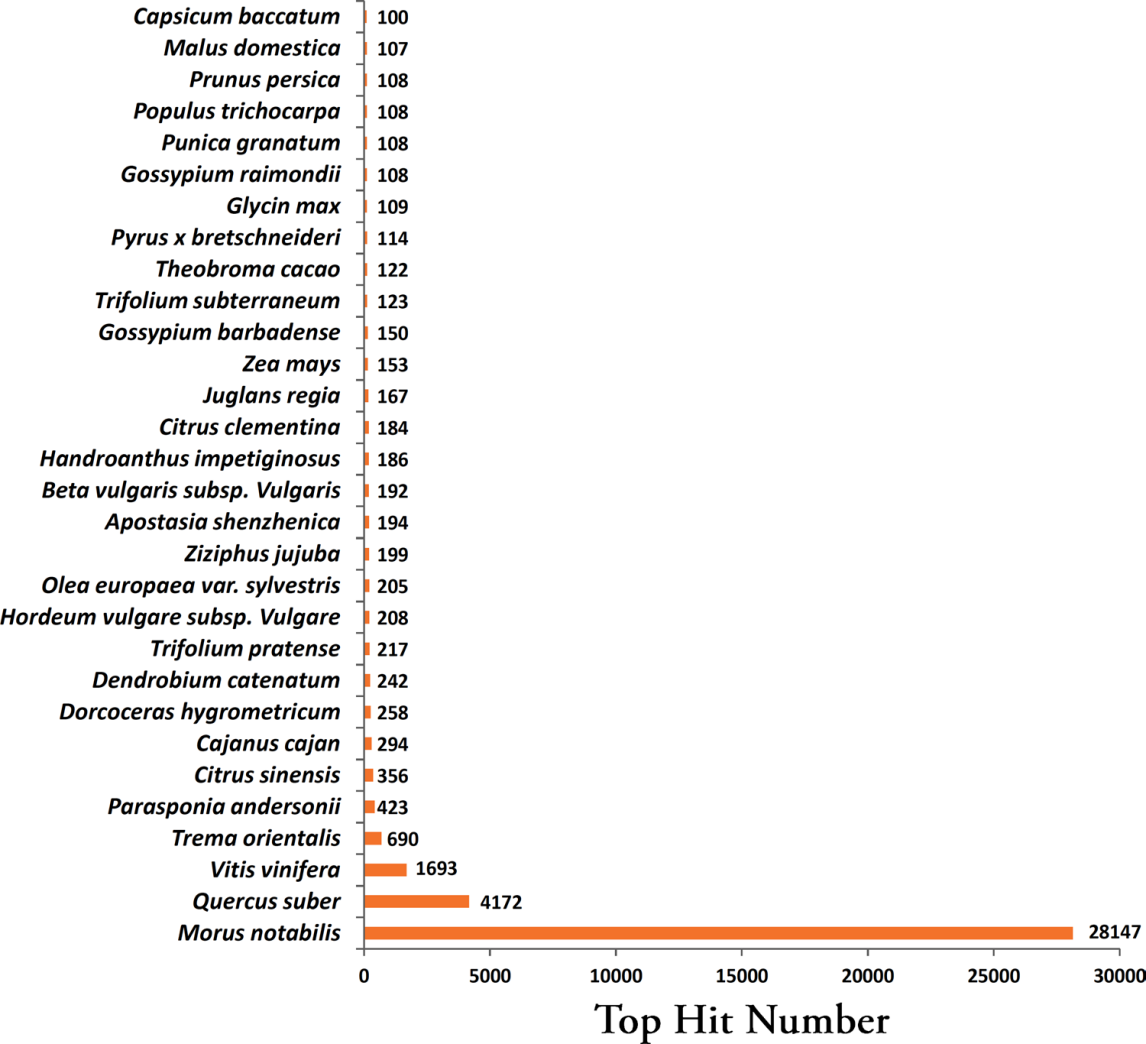
Top hits organism distribution of transcriptome annotation with plantNR database.

**Figure 5:**
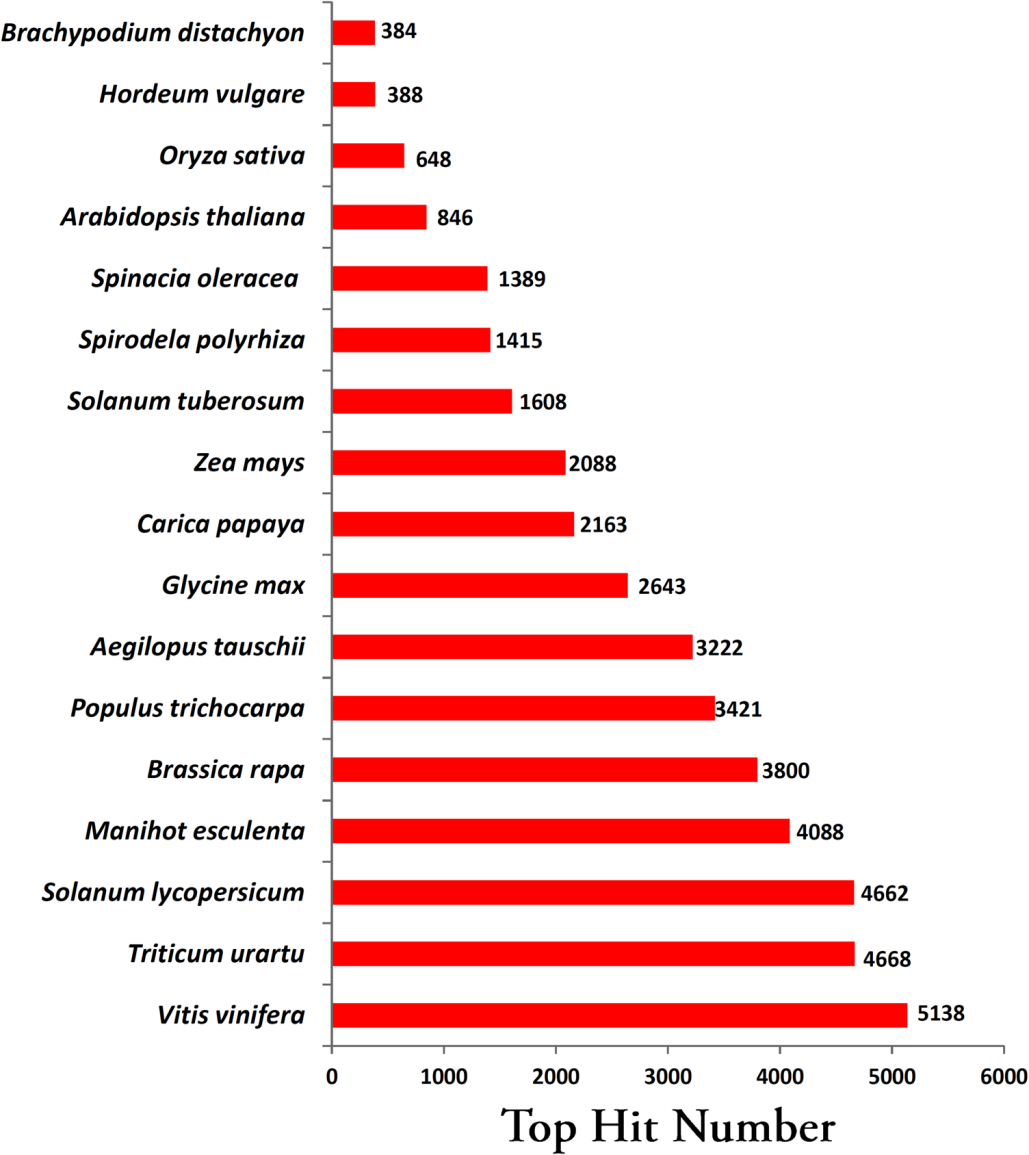
Top hits organism distribution of transcriptome annotation with PMN database.

Distribution of hits across organisms with plantNR database showed maximum similarity with *Morus notabilis* (62.17%), followed by *Quercus suber* (9.21%), and *Vitis vinifera* (3.73%) (Fig. 4). In contrast, transcriptome annotation with the PMN database showed a maximum hit with *Vitis vinifera* (11.06%), followed by *Triticum urartu* (10.05%) and *Solanum lycopersicum* (10.03%) (Fig. 5).

### SSR prediction

**Figure 6:**
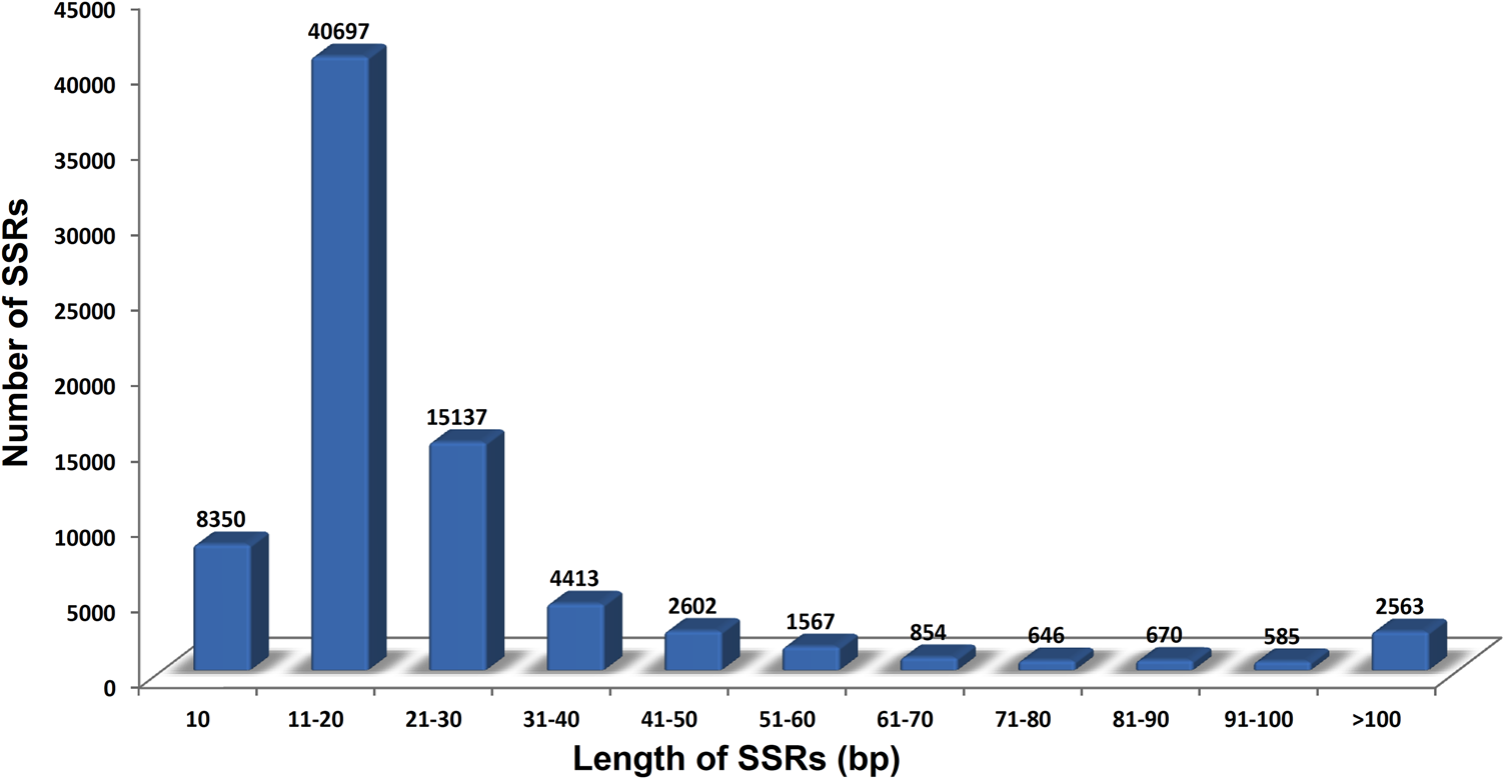
Length distribution of identified SSRs.

The transcripts were operated through the MISA tool for identifying the SSRs (Supplementary Dataset S3). MISA identified 78085 out of 157980 transcripts, of which 67018 contain SSRs (Supplementary Table 3). Out of examined sequences, ~ 30% of transcripts contain more than one SSRs. The obtained summary indicates that SSRs with repeat motifs in the range of 1 to 3 bp of mono-, di- and tri nucleotides repeats accounting ~ 95% of the total identified SSRs (Table 3). In mono-nucleotide repeats, A/T was the most abundant, while G/C was less frequent, accounting for ~ 1% of the total monomer repeats (Table 4). In di-, tri-,tetra- and penta-nucleotides repeats the most abundant type of motif was AG/CT (5901, ~ 31%), TTC/GAA (1233, ~ 14%), AAAT/ATTT (254, ~ 25%), and AAAAT/ATTTT (31, 12%) followed by GA/TC (5275, ~ 28%), TCT/AGA (1031, ~ 12%), TTTA/TAAA (172, ~ 17%), and AAATA/TATTT (29, ~ 11%) while AT/TA (5097, ~ 27%), AAG/CTT (958, ~ 11%), TTAT/ATAA (81, ~ 8%), and TTTTC/GAAAA (25, ~ 10%) occupied the third frequent position respectively. SSRs were classified into 11 groups based on length distribution, maintaining a homogenous gap of 10 units between two groups (Fig. 6). SSRs with 10 bp are the shortest of the lot occupying ~ 10% of the total SSRs. In contrast, SSRs with a base-pair range between 11–20 and 21–30 occupy ~ 52% and ~ 19%, respectively. In SSRs with di-nucleotides having the most abundant repeat length was 12 bp (3424, ~ 18%) followed by 24 bp (2527, ~ 13%) and 22 bp (2277, ~ 12%). Among tri-, tetra-, penta- and hexa-nucleotide repeats, the most abundant repeat length were 15 bp, 20 bp, 25 bp, and 30 bp, respectively. The nucleotide repeats length in penta- and hexa-nucleotide ranged between 25–40 bp and 30–48 bp, respectively.

**Table 3:** Repeat motif based distribution of identified SSRs.

Number of repetitive unit	Number of nucleotide repeats					
	Mono-	Di-	Tri-	Tetra-	Penta-	Hexa-
1	27050	13663	5671	714	190	65
2	8318	3761	2172	214	45	29
3	2186	842	500	48	8	13
4	685	239	119	10	2	0
5	179	73	31	1	1	0
6	64	15	13	1	0	0
7	32	8	2	1	0	0
8	19	3	0	0	0	0
9	9	4	0	0	0	0
10	6	0	0	0	0	0
> 10	9	3	0	0	0	0
Total	38557	18611	8508	989	246	107

**Table 4:** Frequency summary of SSRs with different numbers of tandem repeats.

Repeat type	Count	Repeat type	Count	Repeat type	Count
**Mono-nucleotide**	38556	**Di-nucleotide**	18611	**Tri-nucleotide**	8508
A/T	38106	AG/CT	5901	TTC/GAA	1233
G/C	450	GA/TC	5275	TCT/AGA	1031
		AT/TA	5097	AAG/CTT	958
		TG/CA	1300	AAT/ATT	901
		AC/GT	992	TTA/TAA	580
		CG/GC	46	TTG/CAA	307
				TCA/TGA	287
				TAT/ATA	244
				CCG/CGG	233
				AAC/GTT	224
				ACA/TGT	215
				CAT/ATG	200
				GAT/ATC	188
				GCC/GGC	180
				TGC/GCA	156
				GAG/CTC	154
				CAG/CTG	152
				CGC/GCG	150
				GCT/AGC	142
				CCA/TGG	139
				CAC/GTG	134
				TCC/GGA	122
				GGT/ACC	111
				TCG/CGA	110
				CCT/AGG	94
				TAG/CTA	61
				ACG/CGT	59
				GAC/GTC	58
				ACT/AGT	53
				GTA/TAC	32

### Differential expression analysis (DEG)

**Figure 7:**
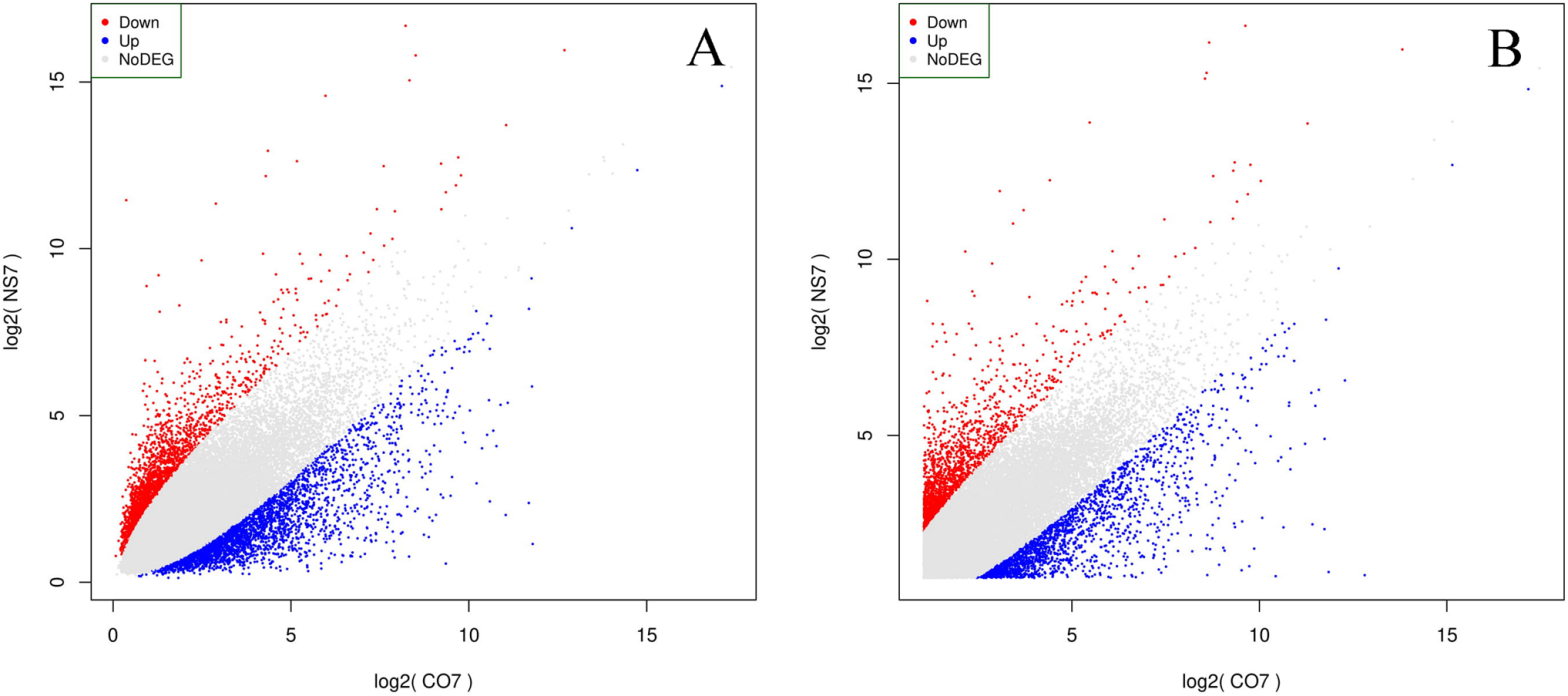
Scatter-plot displaying differentially expressed isoforms (**A**) and unigenes (**B**).

**Figure 8:**
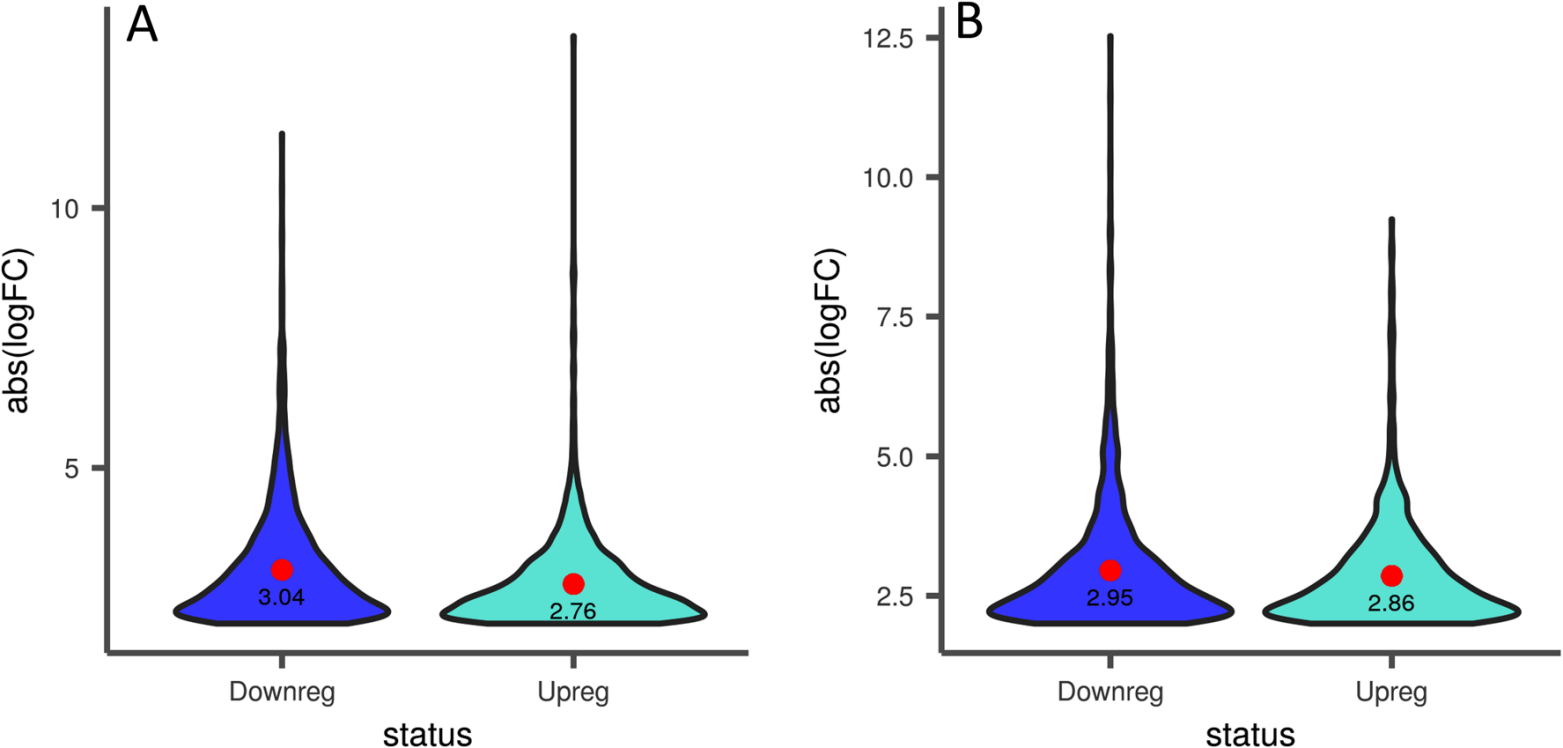
Violin plot representing the log_2_FoldChanges of differentially expressed up-regulated and down-regulated (**A**) isoforms and (**B**) unigenes. The value within the violin indicates average log_2_FoldChange.

**Figure 9:**
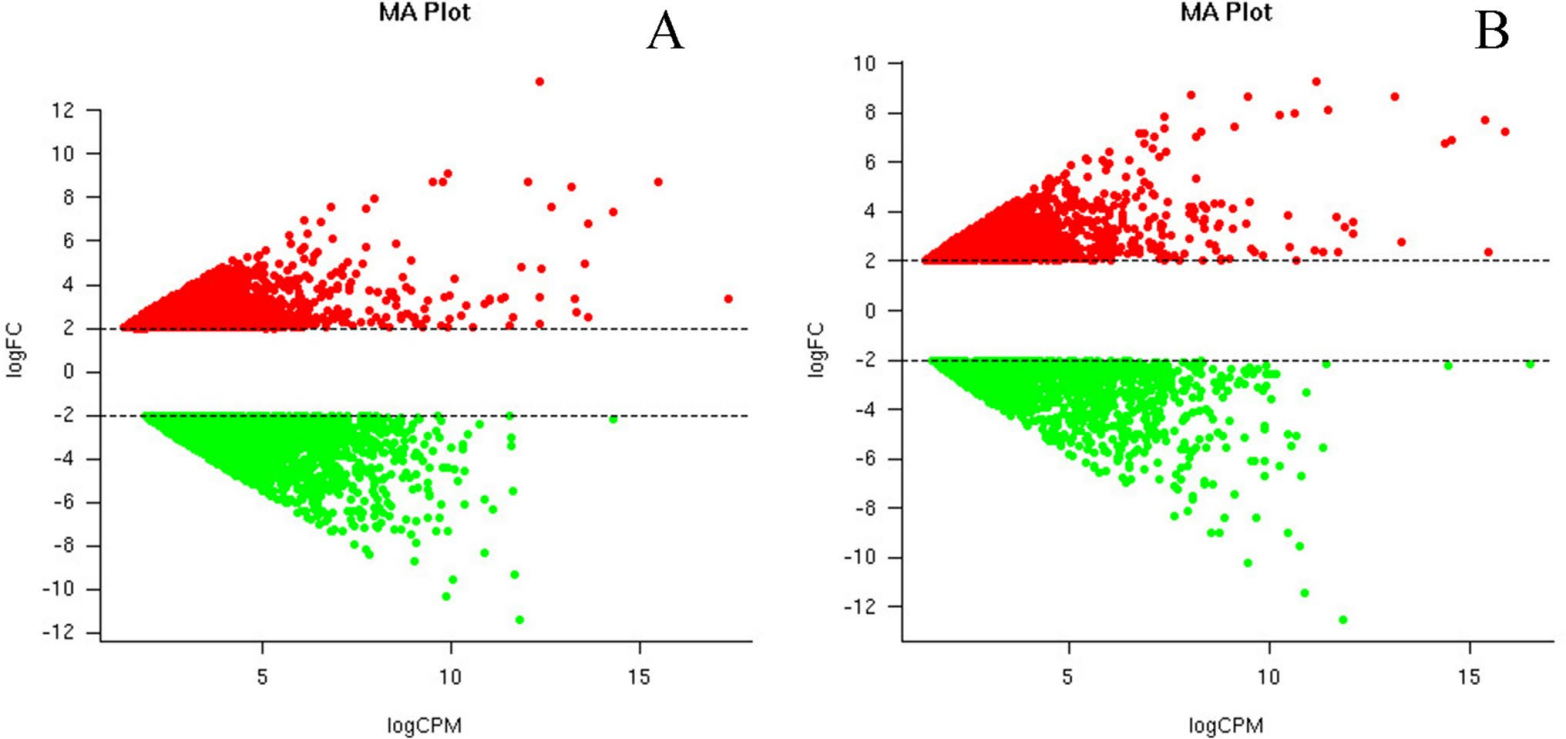
MA plot representing differential expression genes in terms of up-regulated and down-regulated (**A**) isoforms and (**B**) unigenes. The values are represented as the function of logFC vs. logCPM at *p* value of < 0.05 and FDR rate of < 0.05.

**Figure 10:**
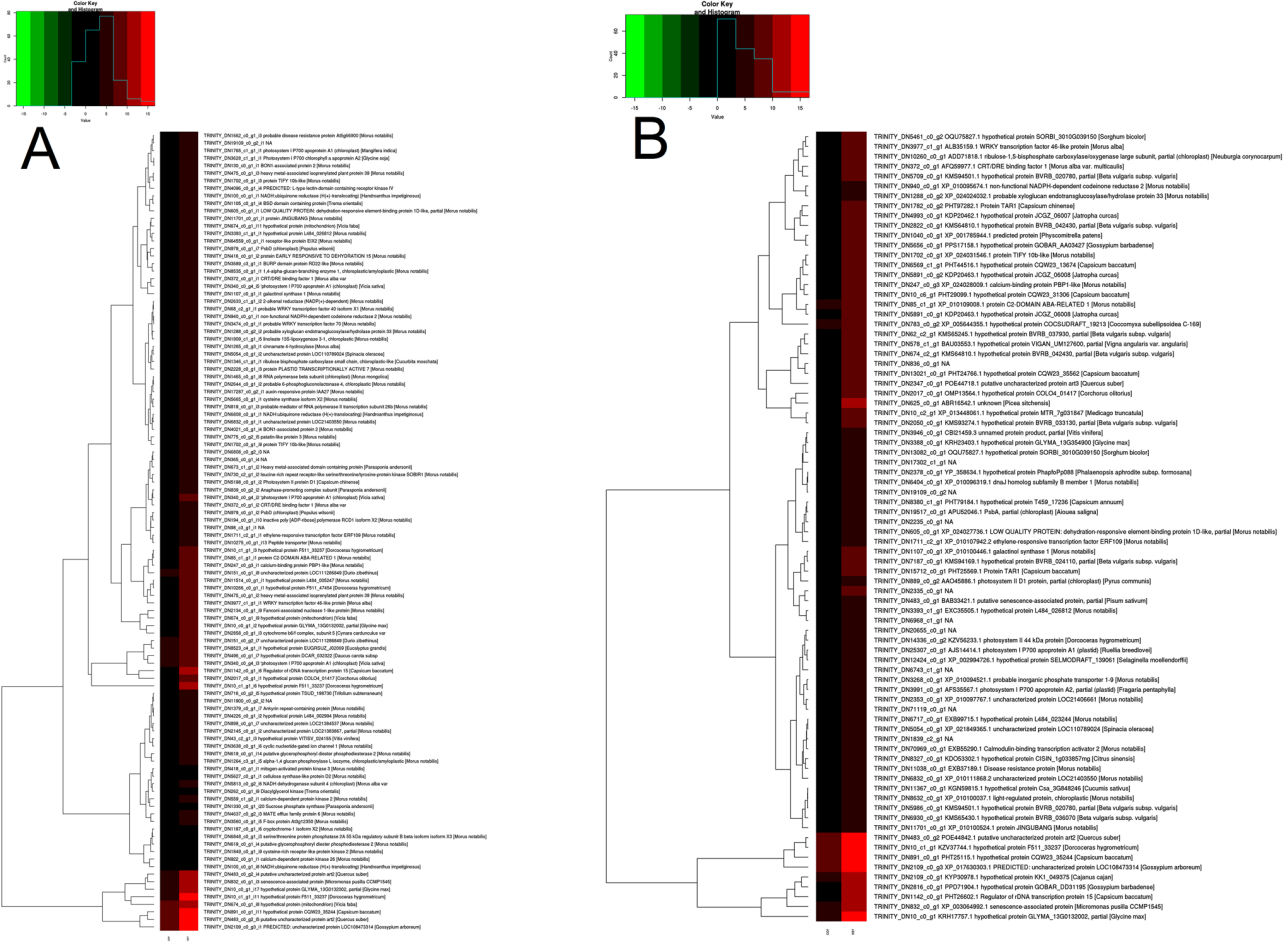
Hierarchical clustering of differentially expressed top 5% up-regulated (**A**) isoforms and (**B**) unigenes in relation to NS7 vs. CO7. The higher score with different colour represents the higher level of expression.

**Figure 11:**
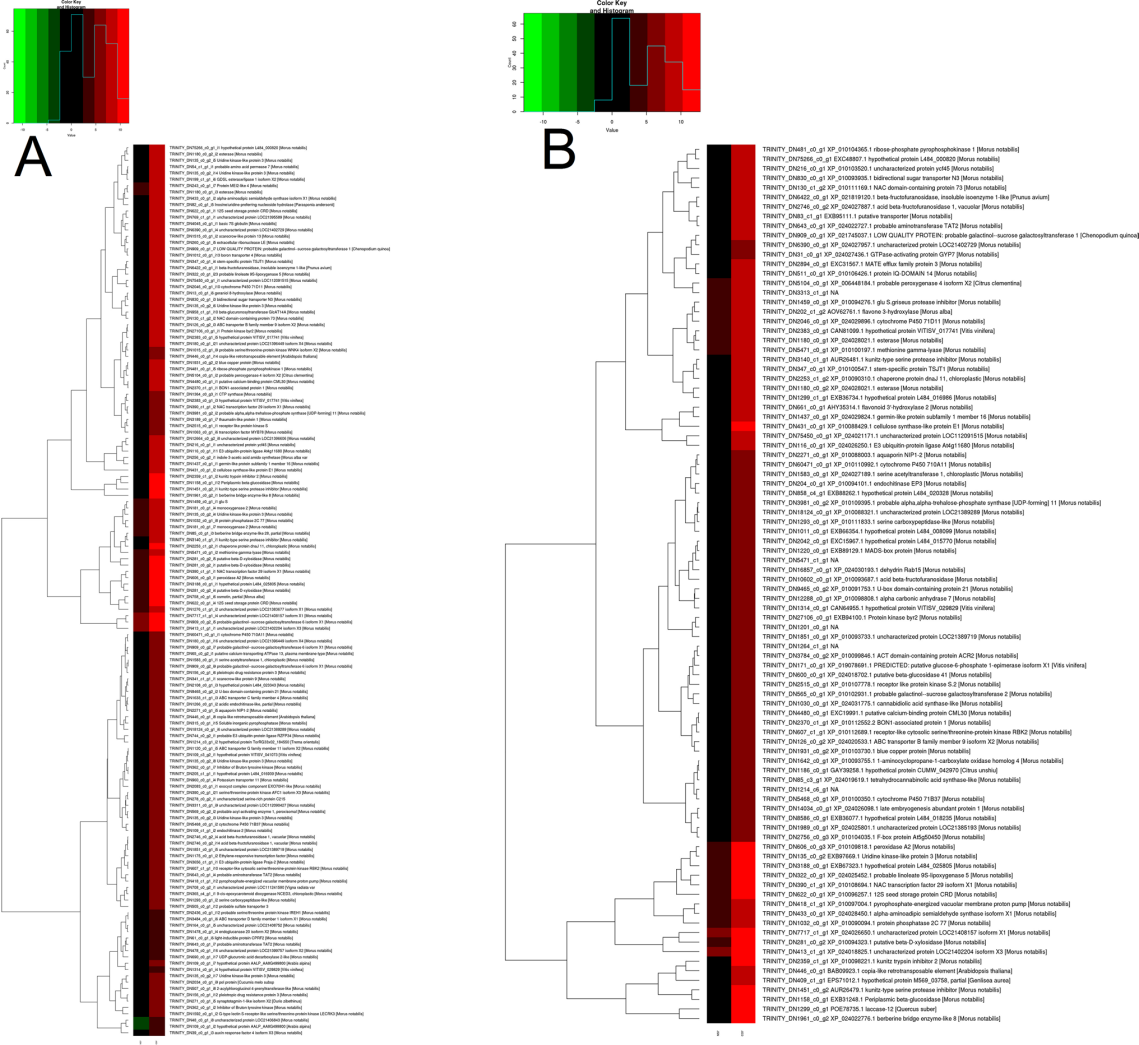
Hierarchical clustering of differentially expressed top 5% down-regulated (**A**) isoforms and (**B**) unigenes in relation to NS7 vs CO7. The higher score with different colour represents the higher level of expression.

The comparative profile analysis was performed with respect to NS7 vs. CO7 for evaluating the post-harvest preservative potentiality of nanosilver (NS) solution. A total of 4918 isoforms and 3413 unigenes were found to be significant differentially expressed, with 2112 isoforms and 1587 unigenes being up-regulated in NS7 and 2806 isoforms and 1826 unigenes being down-regulated in NS7 (Supplementary Table 4). The proportion of differentially expressed unigenes and isoforms are shown using scatter-plot of their expression values (Fig. 7). The up-regulated isoforms had an average log2FoldChange of 2.76 while the down-regulated isoforms had an average log2FoldChange of 3.04. Similarly, the average log2FoldChanges for the up-regulated and down-regulated unigenes were 2.86 and 2.95, respectively (Fig. 8). The complete list of differentially expressed unigenes and isoforms are provided as Supplementary Data S4 and S5. The number of up-regulated and down-regulated isoforms and unigenes were revalidated in terms of MA-plot that takes into account log2FoldChanges in the y-axis versus average expression level in the x-axis (Fig. 9). Red and green dots signifies differentially expressed up-regulated and down-regulated genes with FDR < 0.05. Greater amplitude of differential expression genes occupied extreme data points along the y-axis. In comparison to higher expression value, log2FoldChanges was more variable in lower mean expression values. The volcano plots of expression between the samples lead to the identification of differentially expressed isoforms and unigenes in terms of log2FoldChange and negative log10FDR (Supplementary Fig. 4), indicating the differential expression was highly significant at FDR < 0.05. NCBI Nr database mediated cluster heat map analysis of top 5% up-regulated and down-regulated isoforms and unigenes of the sample comparison at FDR cut-off 0.05 indicates *Morus notabilis* as the top hit organism, which is in support of the above-stated observation (Fig. 10, 11). From heat map analysis, it was noted that proteins associated with photosystem, chloroplast development, defense mechanism, transcription, and cell formation are mainly up-regulated in NS7. Proteins like ion transporters, aquaporin, ABC transporters, cytochrome P450, ubiquitin ligase, and serine-threonine kinase are top down-regulated processes in NS7.

### Functional annotation of differentially expressed up-regulated and down-regulated genes with respect to Arabidopsis database

**Figure 12:**
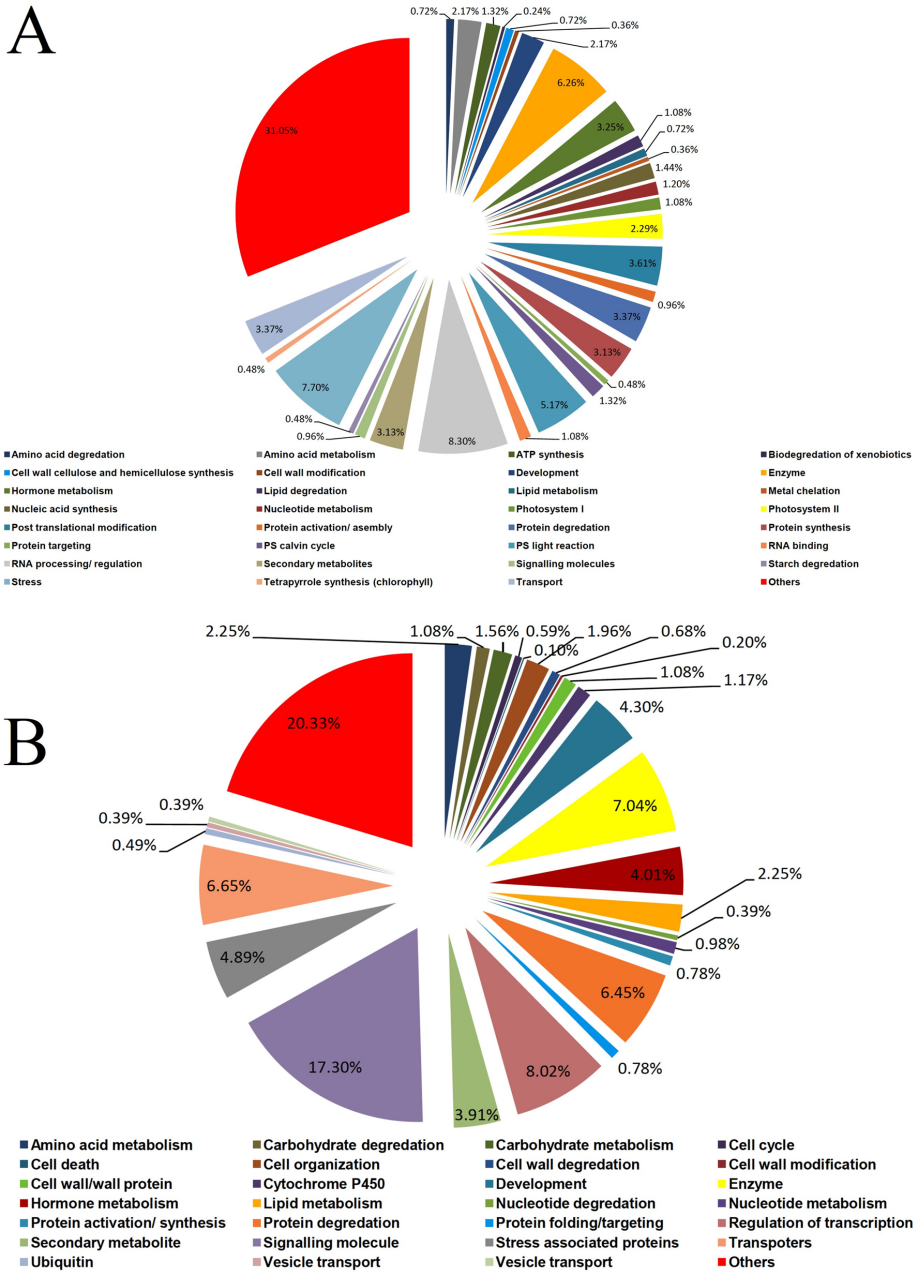
Category wise distribution of differentially expressed (**A**) up-regulated and (**B**) down-regulated genes on annotation with *Arabidopsis* database.

For determining genes of post-harvest shelf-life extension, annotation of differentially expressed (NS7 vs. CO7) up-regulated and down-regulated genes was conducted with the Arabidopsis database. Annotation was done with Mercator (version 3.6) plaBi database that identified 443 up-regulated and 579 down-regulated unigenes in NS7 (Supplementary Dataset S6 and S7). Significantly expressed up-regulated genes remained associated with: photosynthetic processes (~ 10%), processing and regulation of RNA (~ 8%), protein metabolism (~ 8%), stress markers (~ 7.5%), and different enzymatic processes (~ 6%) (Fig. 12A). Besides this, up-regulated gene categories that might play a crucial role in post-harvest self-life elongation are: post-translational protein modification (~ 3.5%), hormone metabolism (~ 3%), secondary metabolites (~ 3%), development associated proteins (~ 2%), ATP synthesis (~ 1%), tetrapyrrole synthesis (~ 0.4%), and biodegradation of xenobiotics (~ 0.2%). Among differentially expressed photosynthetic transcripts, 8% are thylakoid proteins (components of Z-scheme), and ~ 2% are stromal proteins (participates in calvin cycle). Transcripts identified as stress markers are of two types: senescence promoters (~ 2.8%) and senescence inhibitors (~ 4.7%). Senescence promoters are mainly associated with apoptosis-mediated processes. In contrast, senescence inhibitory transcripts were involved in response to pathogens, disease resistance, response to hydrogen peroxide, heat shock proteins (hsp), and light mediated de-etiolation response. *Arabidopsis* database identified down-regulated genes were mostly signalling molecules (~ 18%), transcriptional regulators (~ 8.5%), transporters (~ 7%), stress indicators (~ 5%), and secondary metabolites (~ 4%) (Fig. 12B). Besides this, protein involved in the degradation of carbohydrate, nucleotide, and cell walls were also down-regulated in NS7. Down-regulation of some essential cascade proteins in CO7 (viz., cellular response to water deprivation, the unfolding response of hsp, and ubiquitin-protein ligase activity) indicates low intercellular WHC and high degree of protein denaturation. Events like systemic acquired resistance, innate immune response, apoptosis mediated cellular defence, hypersensitive response were also recorded to be down-regulated, reveling potentiality of NS7 in extending post-harvest shelf life by maintaining cellular integrity and preventing pathogen mediated degradation and blockage of conducting pathways.

### Functional classification and identification of differentially expressed up-regulated and down-regulated genes related to post-harvest shelf-life extension using STRING and Cytoscape

The *Arabidopsis* Gene IDs obtained from the Mercator database were re-verified with the Uniport database, and corresponding Uniport Gene IDs were enlisted. Uniport Gene IDs were used for functional enrichment analysis through protein–protein interaction networks using STRING 11.0. Out of 443 up-regulated and 579 down-regulated genes in NS7, STRING identified 441, 572 nodes and 4651, 4541 edges, respectively, having PPI enrichment of *p* < 1.0e−16 (Supplementary Fig. 5, 6). The average node degree and average local clustering coefficient were recorded to be 21.3, 16, and 0.343, 0.263, respectively. Two rounds of STRING analysis was conducted to screen out top up-regulated and down-regulated unigenes playing a crucial role in extending the post-harvest shelf life in NS7. Besides this, the screening rounds will eliminate less influential genes, which will help to identify genes primarily associated with post-harvest shelf life extension. From the first round of analysis, GO scoring of obtained processes under biological process (BP1), cellular component (CC1), and molecular function (MF1) was first calculated with respect to the function of observed gene count by up/down regulated gene count with STRING background gene count by total *Arabidopsis* background gene count (Supplementary Dataset S8, S9, S10, S11, S12, and S13). Then individual gene scoring was calculated by summating the GO scores of the processes at which a particular unigene was identified by STRING. The unique scoring of the genes was expressed in terms of percentile ranking. Finally, the average percentile ranking was calculated by combining the GO percentile ranking of BP1, CC1, and MF1. The unigenes having cumulative ranking score of 80% and above were selected for downstream analysis. Following the above screening process, 71 up-regulated and 82 down-regulated genes in NS7 were selected and were allowed to pass through round two STRING analyses (Supplementary Dataset S14 and S15). STRING mediated comparison of DEGs of NS7 with respect to CO7 showed significantly enriched GO annotation categories for up-regulated genes. Under BP1 STRING identified major classifiers were: cellular process (GO:0009987), metabolic process (GO:0008152), metabolic process of organic substance (GO:0071704), cellular metabolic process (GO:0044237), primary metabolic process (GO:0044238), and metabolic process of nitrogen compound (GO:0006807). Significantly enriched under CC1 classifiers were: cell and different cell organelles (GO:0005623, GO:0005622, GO:0043226, GO:0043229, GO:0043227, GO:0043231). Whereas top-ranked MF1 were: different binding (GO:0005488) and binding processes like organic cyclic compound binding (GO:0097159), heterocyclic compound binding (GO:1901363), ion binding (GO:0043167), anion binding (GO:0043168), small molecule binding (GO:0036094), and nucleotide binding (GO:0000166) along with catalytic activity (GO:0003824) and transferase activity (GO:0016740) (Supplementary Fig. 7). Enzyme under the class transferases showed maximum number of differentially expressed up-regulated genes followed by oxido-reductases and hydrolases (Supplementary Fig. 8).

Significantly enriched GO annotation categories for down-regulated genes include: metabolic process (GO:0008152), cellular process (GO:0009987), organic substance metabolic process (GO:0071704), cellular metabolic process (GO:0044237), primary metabolic process (GO:0044238), nitrogen compound metabolic process (GO:0006807), response to stimulus (GO:0050896), macromolecule metabolic process (GO:0043170), cellular macromolecule metabolic process (GO:0044260), and biological regulation (GO:0065007) as top GO processes under BP1. Genes of cell (GO:0005623), cell part (GO:0044464), and cellular organelle (GO:0043229, GO:0043227, GO:0043231) represent top CC1. While significant MF1 classifiers includes: genes responsible for catalytic activity (GO:0003824), ion binding (GO:0043167), organic cyclic compound binding (GO:0097159), heterocyclic compound binding (GO:1901363), small molecule binding (GO:0036094), and transferase activity (GO:0016740) (Supplementary Fig. 9). Transferases were the top hit among down-regulated enzymes, followed by oxidoreductases and hydrolases (Supplementary Fig. 10).

**Figure 13:**
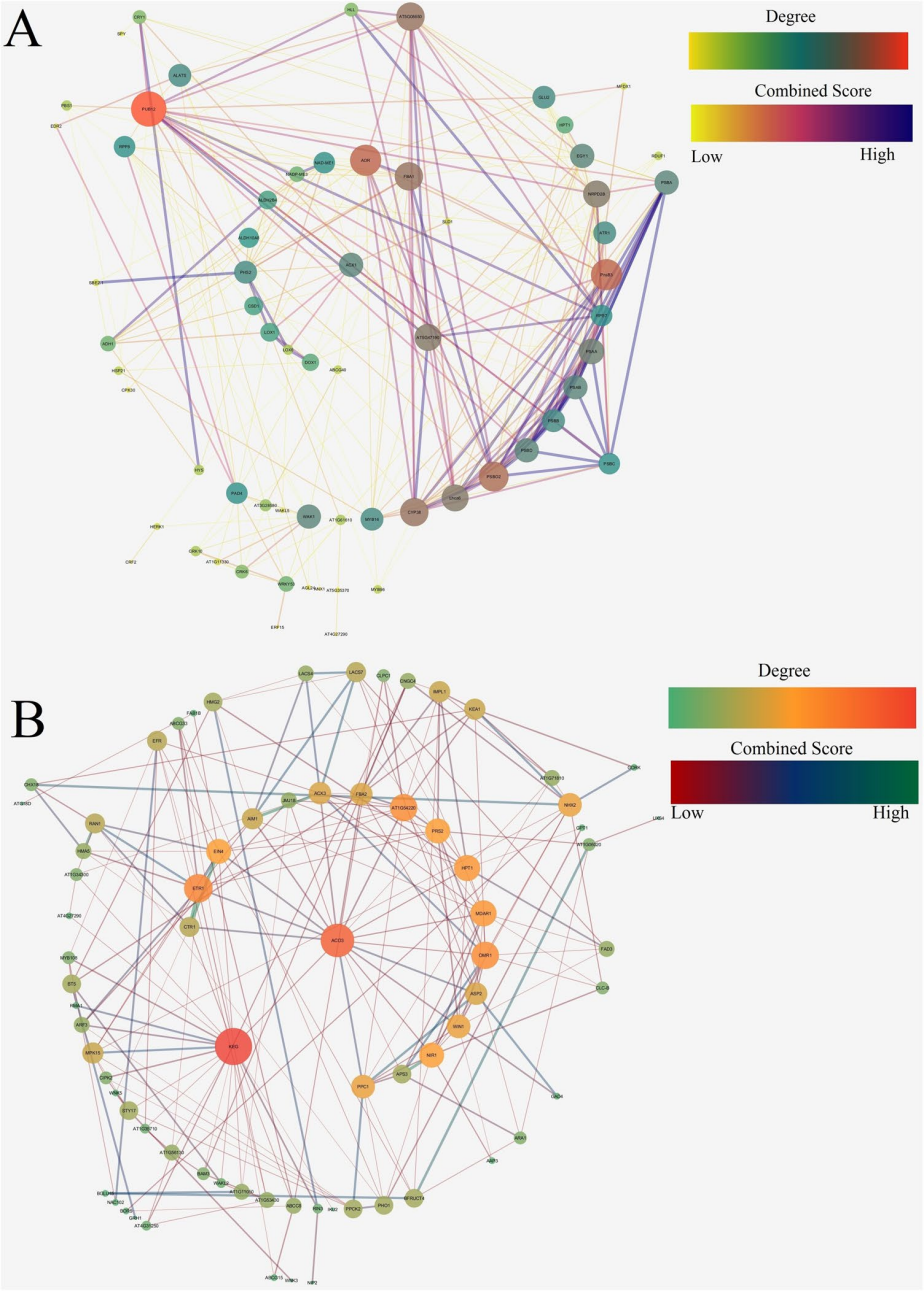
Topological networking of STRING (second round) generated up-regulated (**A**) and down-regulated (**B**) gene interaction data using Cytoscape platform (version 3.7.1; https://cytoscape.org/). For up-regulated genes Cytoscape connected 65 nodes with network density and homogeneity of 0.143 and 0.613 respectively, with characteristic path length of 2.480 and average number of neighbours of 9.169. For down-regulated genes Cytoscape connected 74 nodes with network density and homogeneity of 0.080 and 0.690 respectively, with characteristic path length of 2.785 and average number of neighbours of 5.835.

**Figure 14:**
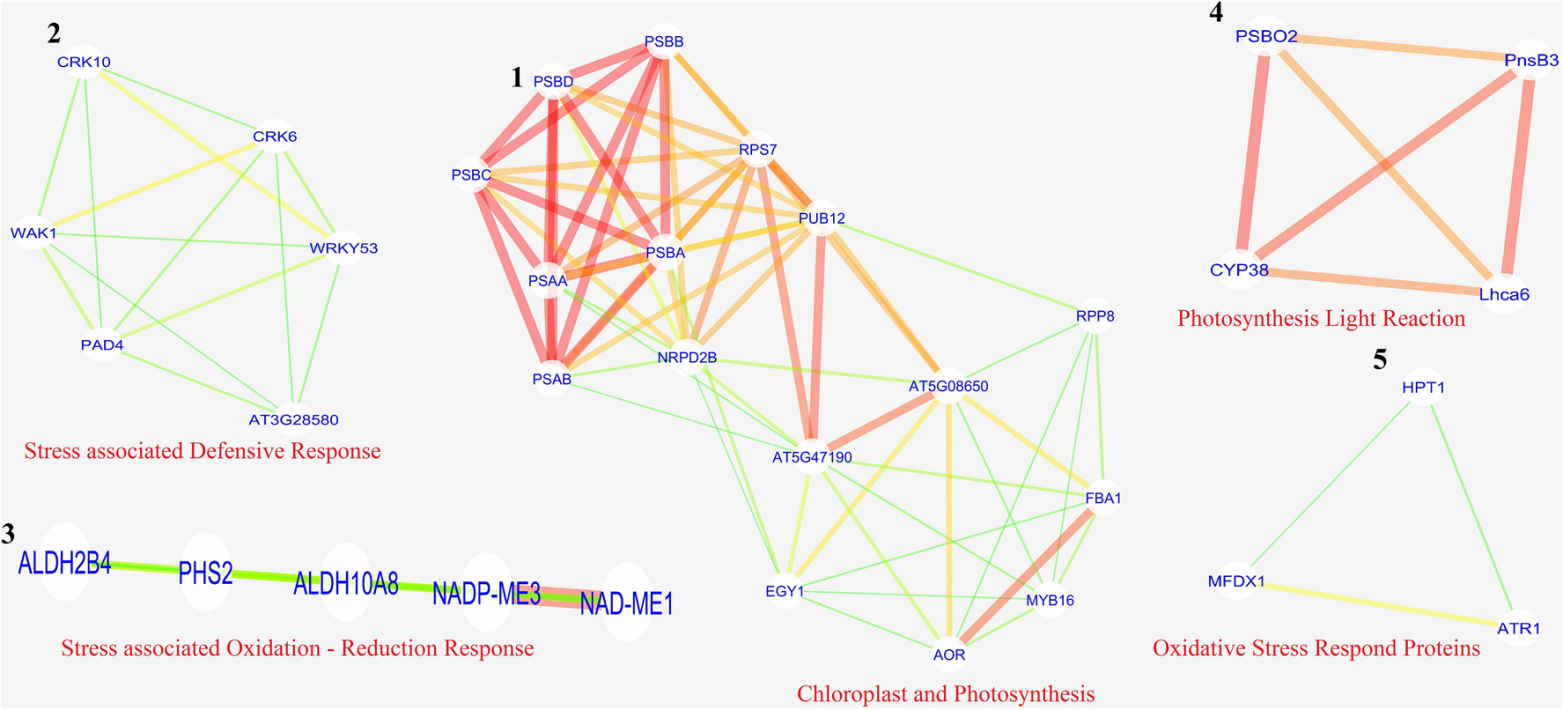
Functional sub-networking of highly interconnected nodes (up-regulated genes) using MCODE (version 2.0.0). Networks were built considering node density and node score cut-off value of 0.1, 0.2 respectively, with kappa-core value of 2.

The second round of STRING analysis with selected up-regulated and down-regulated genes displayed 71, 82 nodes, and 298, 216 edges, respectively, having PPI enrichment of *p* < 1.0e−16 (Supplementary Figs. 11, 12). The STRING interaction data (Supplementary Dataset S16 and S17) for both up-regulated and down-regulated genes were transported to Cytoscape_v3.7.1 for studying the topology. Cytoscape network analysis was constructed using a radial layout concerning degree as map node size and combined score as map edge size. From the STRING interaction network, Cytoscape identified 65, 74 nodes with clustering coefficients of 0.406, 0.316 and network homogeneity of 0.613, 0.690 respectively, for up-regulated and down-regulated genes (Fig. 13). The average topological score was calculated considering: betweenness centrality, closeness centrality, degree, radiality, stress, and Eigenvalue (Supplementary Dataset S18 and S19). From topological study, top hit up-regulated genes were recorded to be associated with: transcriptional process [viz*.* NRPD2B (DNA-directed RNA polymerase D subunit 2b), MYB16 (transcription factor MYB16)]; translational process [viz*.* AT5G47190 (50S ribosomal protein L19-2, chloroplastic), ALATS (alanine-tRNA ligase), AT5G08650 (translation factor GUF1 homolog, chloroplastic), GLU2 (ferredoxin dependent glutamate synthase 2, chloroplastic)]; photosynthetic process [viz*.* PnsB3 (photosynthetic NDH subunit of subcomplex B 3, chloroplastic), Lhca6 (photosystem I chlorophyll a/b-binding protein 6, chloroplastic), PSAA (photosystem I P700 chlorophyll a apoprotein A1), PSAB (photosystem I P700 chlorophyll a apoprotein A2), PSBB (photosystem II CP47 reaction centre protein), PSBD (photosystem II D2 protein), PSBA (photosystem II protein D1), CYP38 (Peptidyl-prolyl cis–trans isomerase, chloroplastic), EGY1 (zinc-metalloprotease, chloroplastic), PSBO2(oxygen-evolving enhancer protein 1–2, chloroplastic)]; carbohydrate metabolism [viz*.* PHS2 (alpha-glucan phosphorylase 2, cytosolic), FBA1 (fructose bisphosphate aldolase), NAD-ME1 (NAD-dependent malic enzyme 1, mitochondrial)]; oxidative stress management [viz*.* DOX1 (alpha-dioxygenase 1), CSD1 (superoxide dismutase), AOR (NADPH dependent alkenal/one oxidoreductase, chloroplastic), ALDH10A8 (betaine aldehyde dehydrogenase 1, chloroplastic)]. Besides this, kinases like WAK1 (wall-associated receptor kinase 1), and PBS1 (serine/threonine-protein kinase PBS1); enzymes like ALDH2B4 (aldehyde dehydrogenase family 2 member B4, mitochondrial), ATR1 (NADPH cytochrome P450 reductase 1), and ACX1 (peroxisomal acyl-coenzyme A oxidase 1) are also recorded to be up-regulated. On comparing up-regulated genes with respect to the magnitude of expression (NS7 vs. CO7), it was observed that photosynthetic genes showed the highest magnitude of difference in expression pattern with PSBB, PSAB, PSAA showed more than 20-fold, and PSBA, PSBD, PHS2 showed less than 20-fold expression profile (Supplementary Fig. 13). Five sub-networks were identified using MCODE (v_2.0.0) based on highly interconnected nodes (Fig. 14). Sub-network 1 and 4 comprise 16 (PSBC, EGY1, RPS7, AOR, PSBB, AT5G08650, PSBD, PSBA, PUB12, FBA1, PSAA, RPP8, PSAB, AT5G47190, NRPD2B, and MYB16), and 4 (PnsB3, PSBO2, Lhca6, and CYP38) nodes interconnected with 66, and 6 edges having node density of 8.8, and 4, associated with photosynthetic processes. Sub-network 2, 3, and 5 forms clustering with 6 (WRKY53, AT3G28580, WAK1, CRK10, PAD4, and CRK6), 5 (NAD-ME1, ALDH2B4, ALDH10A8, NADP-ME3, and PHS2), and 3 (MFDX1, HPT1, ATR1) nodes linked among each other by 14, 10, and 3 edges were having node density of 6, 5, and 3 respectively, associated with stress management.

**Figure 15:**
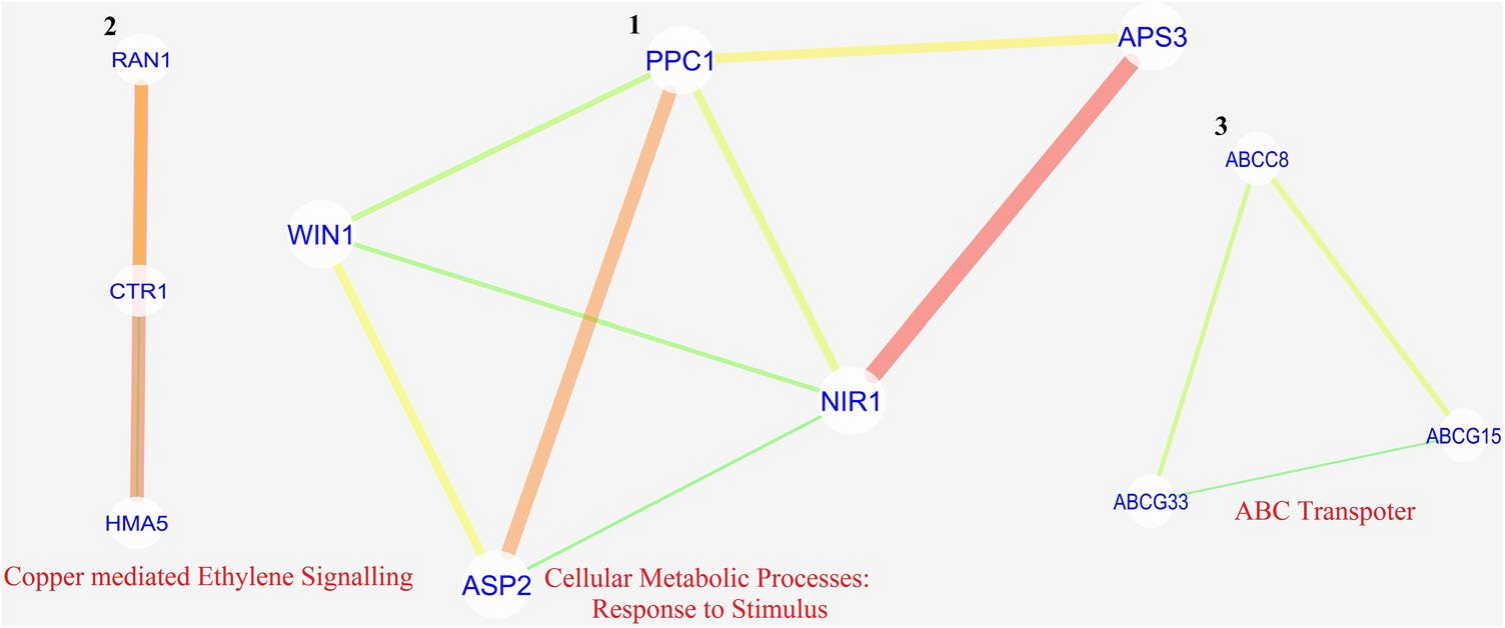
Functional sub-networking of highly interconnected nodes (down-regulated genes) using MCODE (version 2.0.0). Networks were built considering note density and node score cut-off value of 0.1, 0.2 respectively, with kappa-core value of 2.

Top hit down-regulated genes obtained from topological analysis remained associated with: sucrose mobilization [viz*.* BFRUCT4 (acid beta-fructofuranosidase 4, vacuolar)]; plant hormone [viz*.* ETR1 (ethylene receptor 1), EIN4, CTR1 (serine/threonine-protein kinase), and ARF3 (auxin response factor 3)]; transporters/channels [viz*.* NHX2 (sodium/hydrogen exchanger 2), KEA1 (K + efflux antiporter 1, chloroplastic), RAN1 (copper transporting ATPase), ABCC8 (ABC transporter C family member 8), PHO1 (phosphate transporter), and CNGC4 (cyclic nucleotide-gated ion channel 4)]; ubiquitination process [viz*.* KEG (E3 ubiquitin-protein ligase), BT5 (BTB/POZ and TAZ domain-containing protein 5)]; ammonia production process [viz*.* NIR1 (ferredoxin-nitrite reductase, chloroplastic), and OMR1 (threonine dehydratase biosynthetic, chloroplastic)]; beta-oxidation [viz*.* AIM1 (peroxisomal fatty acid beta-oxidation multifunctional protein), and ACX3 (acyl-coenzyme A oxidase 3, peroxisomal)]; protein associated with kinase activity [viz*.* PRS2 (ribose-phosphate pyrophosphokinase 2, chloroplastic), MPK15 (mitogen-activated protein kinase 15), PPCK2 (phosphoenolpyruvate carboxylase kinase 2), STY17 (serine/threonine-protein kinase) and EFR (LRR receptor-like serine/threonine-protein kinase)]; primary metabolic process [viz*.* PPC1 (phosphoenolpyruvate carboxylase 1), and FBA2 (fructose-bisphosphate aldolase 2, chloroplastic)]; oxidative stress management [viz*.* ACO3 (aconitate hydratase 3, mitochondrial), MDAR1 (monodehydroascorbate reductase 1, peroxisomal), and HPT1 (homogentisate phytyltransferase 1, chloroplastic)]; secondary metabolite synthesis [viz*.* HMG2 (3-hydroxy-3-methylglutaryl-coenzyme A reductase 2)]; intracellular sulphate activation [viz*.* APS3 (ATP-sulfurylase 3, chloroplastic)]. Besides these phosphatase like IMPL1; aminotransferase like WIN1 (acetylornithine aminotransferase, chloroplastic/mitochondrial), and ASP2 (aspartate aminotransferase, cytoplasmic isozyme 1) are also down-regulated. Concerning magnitude of expression (NS7 vs. CO7), BFRUCT4 and PRS2 showed more than 50-fold expression, while CNGC4, PHO1, and APS3 depict expression in the range between 50–20 fold, and the remaining has less than 20-fold expression profile (Supplementary Fig. 14). MCODE mediated determination of sub-network identified three major interaction patterns (Fig. 15). Cluster 1 has five nodes (ASP2, PPC1, NIR1, APS3, and WIN1) connected with eight edges, associated with cellular metabolism, while cluster 2 and 3 contains three nodes (HMA5, CTR1, and RAN1; ABCG15, ABCC8, ABCG33) connected with three edges each, associated with ethylene signaling and ABC transport system respectively.

### BiNGO and KEGG based functional categorization of differentially expressed transcripts

**Figure 16:**
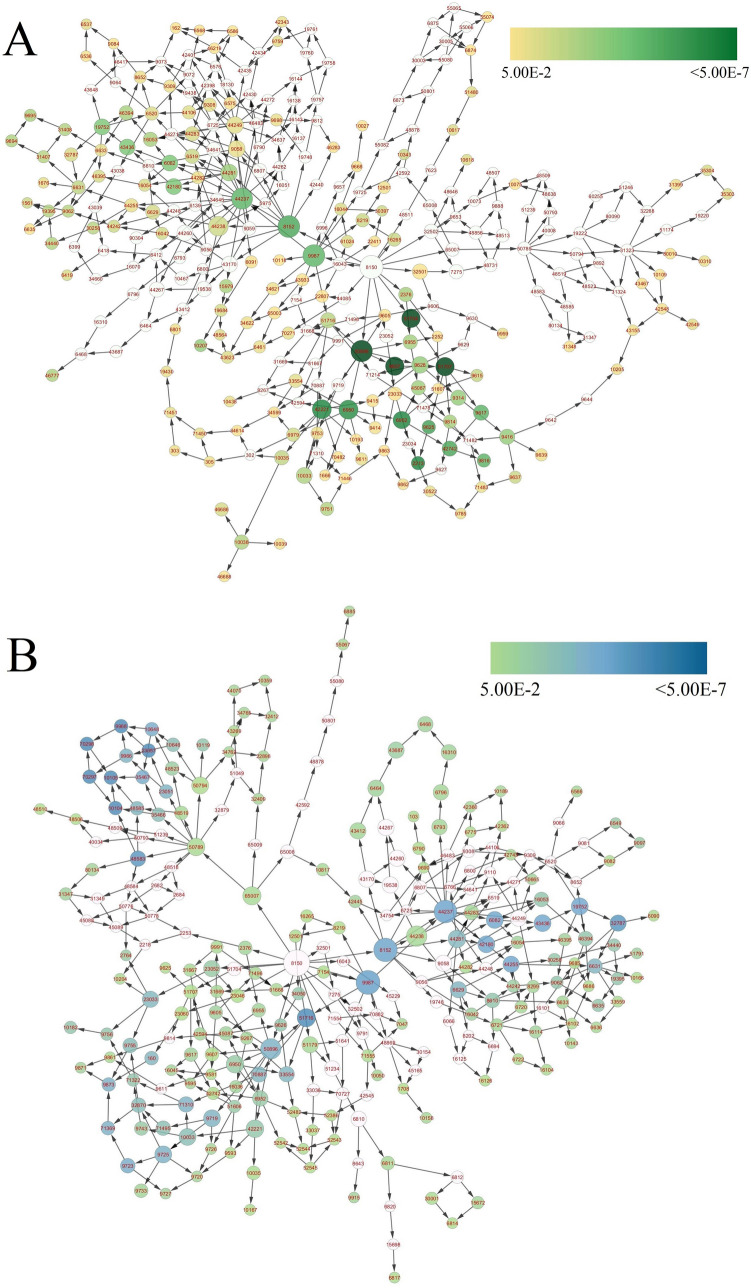
Gene ontological enrichment analysis of topologically selected up-regulated (**A**) and down-regulated (**B**) genes based on biological process using BiNGO (version 3.0.4) considering *Arabidopsis* as model organism. Hypergeometric test was conducted, considering *p* value cut off ≤ 0.05. Size of the node was proportional to the number of gene (transcripts) present under a particular nodal category. Node colour shades were according to the significance level. For up-regulated genes white represents no significant differences, yellow and green colour shade represents significance level at *p* = 0.05 and < 0.0000005 respectively. Similarly for down-regulated genes white represents no significant differences, green and blue colour shade represents significance level at *p* = 0.05 and < 0.0000005 respectively.

BiNGO (version 3.0.4) mediated enrichment analysis of topologically selected differentially expressed transcripts was carried out, which has crossed the 50% average percentile mark. BiNGO will help understand the involvement of various biological processes in extending the shelf-life of post-harvest preserved mulberry leaves. GO-based BiNGO study identified 157 and 182 processes under up-regulated and down-regulated differentially expressed transcripts in NS7, out of which 43 processes were found to be similar (Supplementary Dataset S20 and S21). Processes like: cellular ketone metabolic process, defense response, defense response to bacterium, fatty acid beta-oxidation, immune response, mono-carboxylic acid metabolic process, organic acid metabolic process, oxoacid metabolic process, primary metabolic process, programmed cell death, response to chemical stimulus, response to stress, small molecule metabolic process were found to be shared among the differentially expressed up-regulated and down-regulated genes. Specific up-regulated processes includes: anthocyanin metabolic process, blue light signaling pathway, cellular response to blue light, cellular response to oxidative stress, cellular response to salicylic acid stimulus, cellular response to superoxide, photosynthesis (light reaction), photo-systems (I and II assembly), photosystem II stabilization, regulation of hydrogen peroxide (metabolic process), regulation of oxygen and reactive oxygen species (metabolic process), regulation of photosynthesis, removal of superoxide radicals, response to blue light, response to light stimulus, response to oxidative stress, response to oxygen levels, response to oxygen radical, response to red or far-red light, response to superoxide, superoxide metabolic process, thylakoid membrane organization (Fig. 16A). While notable specific down-regulated processes were: cellular response to ethylene stimulus, cellular response to nutrient levels, cellular response to starvation, cellular response to stress, detection of ethylene stimulus, fat-soluble vitamin metabolic process, gibberellin metabolic process, hormone-mediated signaling pathway, hydrogen peroxide metabolic process, phloem loading, plant-type hypersensitive response, regulation of defense response, regulation of anion channel activity, regulation of ethylene mediated signaling pathway, regulation of pH, vitamin E biosynthetic process, and vitamin E metabolic process (Fig. 16B).

**Figure 17:**
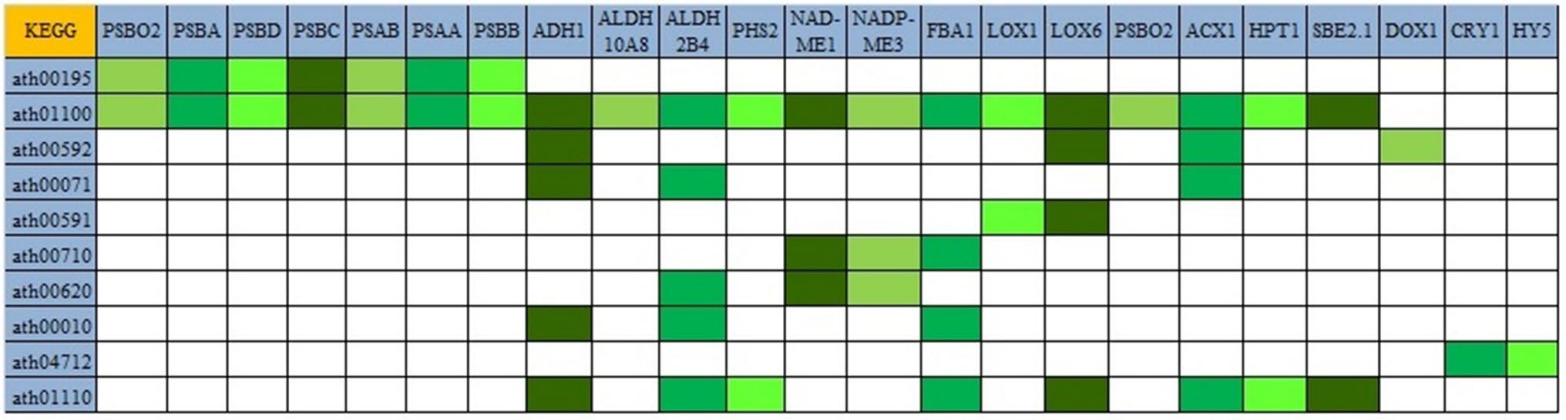
Kyoto Encyclopaedia of Genes and Genomes (KEGG) classification of top differentially expressed (up-regulated) genes. The genes were enlisted at the top and the colour pattern (green shades) indicates the involvement of a particular gene in a particular KEGG pathway.

**Figure 18:**
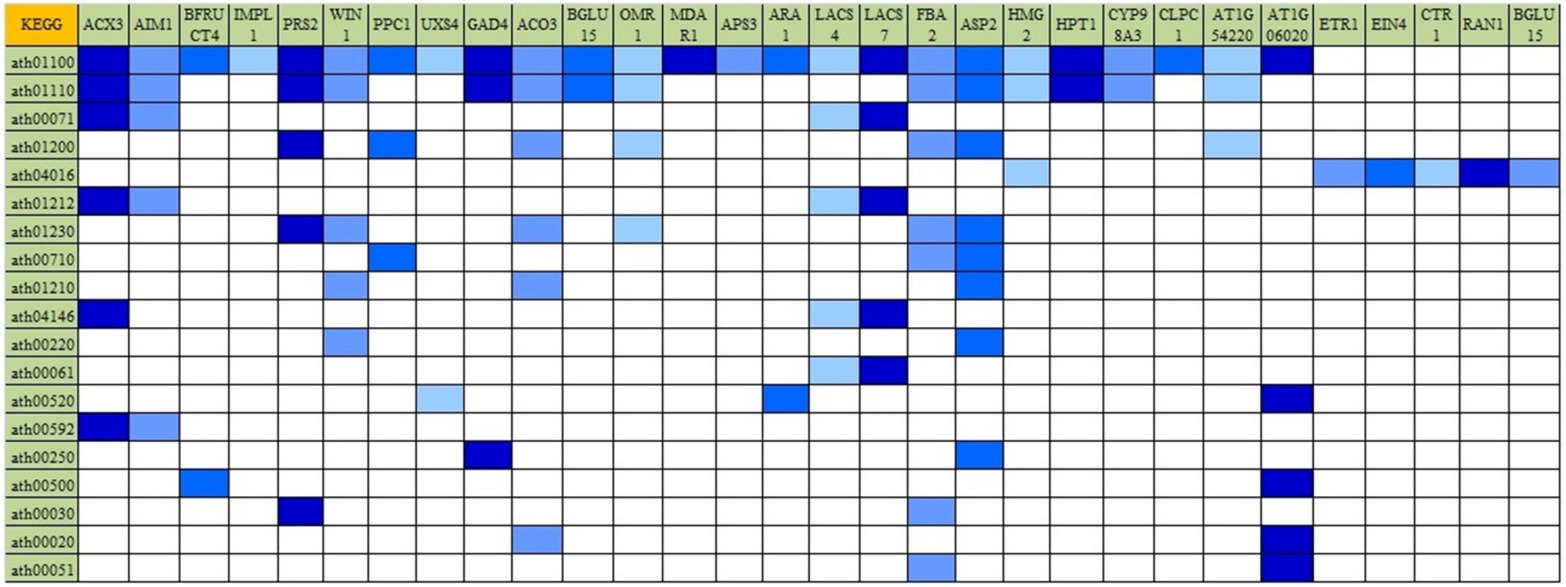
Kyoto Encyclopaedia of Genes and Genomes (KEGG) classification of top differentially expressed (down-regulated) genes. The genes were enlisted at the top and the colour pattern (blue shades) indicates the involvement of a particular gene in a particular KEGG pathway.

Screened unigenes were mapped using KEGG to determine the enriched pathways in NS7 and CO7. A total of 10 up-regulated KEGG pathways were identified in NS7, containing 53 annotated sequences (Fig. 17). The identified up-regulated KEGG pathways include: photosynthesis (ath00195), metabolic pathways (ath01100), alpha-linolenic acid metabolism (ath00592), fatty acid degradation (ath00071), linoleic acid metabolism (ath00591), carbon fixation in photosynthetic organisms (ath00710), pyruvate metabolism (ath00620), glycolysis/gluconeogenesis (ath00010), circadian rhythm-plant (ath04712), and biosynthesis of secondary metabolites (ath01110). KEGG identified 19 down-regulated pathways in NS7 containing 94 annotated sequences (Fig. 18). The pathways includes: metabolic pathways (ath01100), biosynthesis of secondary metabolites (ath01110), fatty acid degradation (ath00071), carbon metabolism (ath01200), MAPK signalling pathway-plant (ath04016), fatty acid metabolism (ath01212), biosynthesis of amino acids (ath01230), carbon fixation in photosynthetic organisms (ath00710), 2-oxocarboxylic acid metabolism (ath01210), peroxisome (ath04146), arginine biosynthesis (ath00220), fatty acid biosynthesis (ath00061), amino sugar and nucleotide sugar metabolism (ath00520), alpha-linolenic acid metabolism (ath00592), alanine, aspartate and glutamate metabolism (ath00250), starch and sucrose metabolism (ath00500), pentose phosphate pathway (ath00030), TCA cycle (ath00020), and fructose and mannose metabolism (ath00051).

### Validation of differentially expressed genes by quantitative real-time PCR

Considering topology, expression profile, and KEGG, a sub-set of 8 differentially expressed (4 up-regulated and 4 down-regulated) genes were selected for quantitative real-time PCR analysis using SYBR Green Chemistry. Different genes for real-time PCR were selected from different functional categories. Selected up-regulated genes were PABB, CSD1, PSBO2, and AOR. Selected up-regulated genes were associated with: PSII reaction center protein (CP47), superoxide dismutase (an oxidative stress inhibiting protein), oxygen-evolving enhancer protein in the chloroplast (involved in abiotic stress management), and NADPH-dependent oxidoreductase (involves in eliminating generated ROS) respectively.

**Figure 19:**
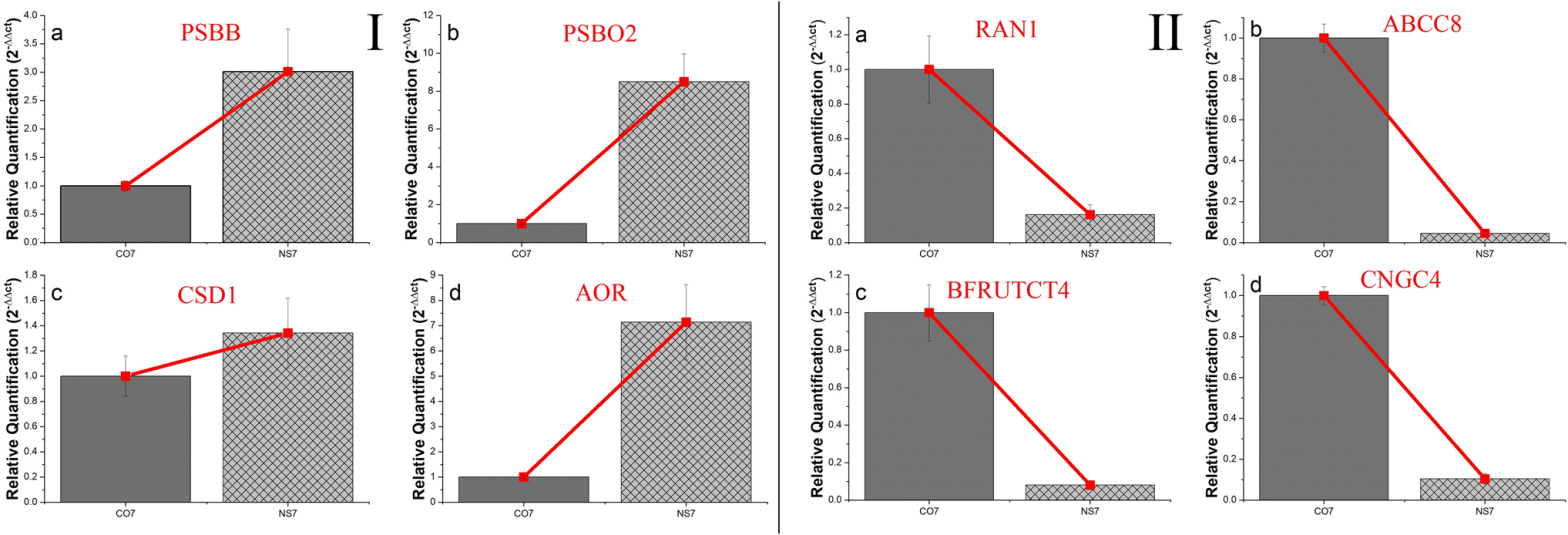
Validation of key differentially expressed up-regulated (**I**) and down-regulated (**II**) genes using qRT-PCR analysis. The y-axis indicates relative quantification of the genes and the studied genes were indicated in the x-axis. Error bar indicates the value of standard deviation (± SD).

The selected down-regulated genes were RAN1, ABCC8, BFRUCT4, and CNGC4. Down-regulated genes were identified to be associated with: copper-transporting ATPase (involved in copper mediated ethylene response), C family of ABC transporter (involved in the regulation of toxic substances generated during yellowing of leaves), acid beta-fructofuranosidase 4 (involved in the mobilization of sucrose to sink organs), and cyclic nucleotide-gated ion channel 4 (involved in hypersensitive response against pathogen mediated defense) respectively. The primer used during RT-PCR amplification was listed in Supplementary Table 5. The real-time PCR results of the differentially expressed genes were in good agreement with the expression profile of transcriptome analysis, suggesting the transcriptome data used in the present study were reliable and accurate (Fig. 19).

## Discussion

Effective preservation of mulberry leaves at the post-harvest stage will significantly impact the global growth of the silk industry in two aspects. Firstly, it will promote the engagement of landless farmers, and secondly, it will increase the rearing productivity during the rainy season. Our earlier study in this aspect identified phytosynthesized silver nanoparticles at 6 ppm concentration significantly retains the physical texture of leaves till day 7 post-harvest^14^. We reported that nanosilver delays the senescence process by preventing the accumulation of molecules capable of causing oxidative damage through up-regulation of defensive processes, thereby preventing deterioration of primary metabolites^13^. The current study attempted to identify novel genes responsible for extending the post-harvest shelf life of mulberry leaves upon nanosilver application with respect to leaves preserved in distilled water for the same duration.

Transcriptome analysis using the Illumina platform is a handy and economical tool for generating information on genes and associated sequences. Illumina-based transcriptome assembly, although based on short read sequencing technology, generates a wide range of transcriptome coverage helpful in identifying genes involved in different processes^26^. The current study using the Illumina platform (Hiseq 4000) generated ~ 191 million raw reads among the studied treatments of mulberry (NS7 and CO7), out of which ~ 71% of high-quality reads (base score ≥ 30) were used for downstream analysis. The quantity of obtained high-quality reads remained within the bracket area of reads required for analyzing differential expression^27^. The obtained N50 value of the assembled transcripts (> 2000) with median contig length > 500 indicates a good QC report of the sequencing data for downstream analysis^28^. The N50 value reported in the current study was almost similar to the N50 value reported for *Morus laevigata* (2086) and *Morus serrata* (2140)^29^. On vetting out transcripts ≤ 200 bp, trinity identified 157982 assembled transcripts and 81952 unigenes, which was in good agreement with earlier studied mulberry transcripts^30,31^. The assembled transcripts showed ~ 55% BLAST hit with plant NR database, which was significantly greater than previous transcript studies on mulberry (*Morus alba*) that showed 41.08% hit^32^. NR database hit was supported by PMN hit, which detected ~ 56% unigenes. Previously studied other *Morus* spp. showed 52% (*Morus laevigata*), 53% (*Morus serrata*) and 59% (*Morus indica*) and 67% (*Morus atropurpurea*) NR BLAST hit^17,29,33^. Transcriptome analysis within the members of the family Moraceae showed 76% (*Artocarpus heterophyllus*), 74% (*Ficus carica*), and 47% (*Ficus hirta*) NR BLAST hit^34–36^. Members of the order Rosales depicted 71% (*Pyrus communis*, Rosaceae), 76% (*Humulus lupulus*, Cannabaceae), 54% (*Ziziphus jujube*, Rhamnaceae), 46% (*Boehmeria nivea*, Urticaceae), and 41% (*Rubus idaeus*, Rosaceae) NR BLAST hit^24,25,37–39^. Analysis of distributional similarity of the assembled transcripts to other plant species concerning the Nr database showed maximum homology with *Morus notabilis* (~ 62%), indicating mapping of most of the transcript with *Morus notabilis* draft genome. A similar observation was reported by Wang et al.^30^, and Dai et al.^17^ while studying De novo assembly in *Morus alba,* and *Morus atropurpurea*, respectively. Besides *Morus notabilis* which belongs to the family Moraceae, *Morus alba* transcripts in the current study also showed sequence similarity with *Quercus suber* (Fagaceae), *Vitis vinifera* (Vitaceae), and *Trema orientalis* (Cannabaceae). Moraceae and Cannabinaceae come under the order Rosales, whereas Fagaceae and Vitaceae come under the order Fagales, and Vitales, respectively. Evolutionary, the order Rosales, Fagales, and Vitales are considered as sister clade^40,41^, thus transcriptome data correlates significantly with the evolutionary relationship among the sequence identical species, with *Morus notabilis* being the closest relative of *Morus alba*. The sequence similarity index indicates ~ 60% of the assembled unigenes mapped more than 80% of their length, ~ 31% mapped within 80–50% of their length, while the remaining ~ 8% displayed mapped similarity of less than 50%. A similar result was reported by Zhou et al.^42^ while studying the De novo assembly of *Taxus* sp.

The present study also deals with identifying SSR markers, most frequently utilized for developing a cost-effective marker system helpful for studying genetic diversity, genetic structuring, demography, relatedness, and the development of linkage maps. The number of SSR obtained in the current study was much higher than that was reported earlier for *Morus alba* (31799)^31^. SSR count on a grander scale than other species of the family Moraceae viz*., Morus multicaulis* (33285), *Morus laevigata* (12206) and *Morus serrata* (11843)^29,43^. At least 1 SSR was documented in most of the studied transcripts. Most abundantly found transcript types were tri-nucleotide followed by di-nucleotide, which was in accordance with the observation of Victoria et al.^44^, who stated that tri-nucleotide repeats were most frequent in higher plants (angiosperms). In contrast, di-nucleotide repeats were predominant in the lower group of plants. The high proportion of mono-nucleotide repeats may also be due to the errors in NGS sequencing technology^45^. SSRs with A/T motif (~ 98%) were the most abundant mono-nucleotide repeat, while in di-nucleotide AG/CT (~ 31%) leads the ranking, similar to the report of Park et al.^46^ on *Lychnis kiusiana*. AAAT/ATTT (~ 25%) and AAAAT/ATTTT (~ 12%) were the most frequent tetra-, and penta- nucleotide repeat, a similar observation was displayed by Tulsani et al.^47^ and Li et al.^38^ while performing SSR analysis of *Coriandrum sativum* and *Ziziphus jujuba*. SSRs with long repeats are considered highly polymorphic^48^, the present study showed SSRs with length range between 11–20, 21–30, and 31–40 bp chronologically display a greater number of SSRs. The variation in length among the repetitive units was probably because different sequences may have appeared in different generations during evolution^49^. The frequency of occurrence of repetitive units decreases as the number of nucleotides increases. Cho et al.^50^ stated that the appearance of repetitive motifs within plant genome and their length distribution resulted from applied selection pressure during evolution.

The preservative potentiality of an effective preservative solution depends on its ability to retain primary metabolites and prevent the accumulation of compounds responsible for elevating the process of senescence. Among the primary metabolites, chlorophyll and photosynthetic apparatus act as effective marker systems for assisting the effectiveness of the preservative solution. Changes in chlorophyll content below optimum level were an indicative symbol of plant stress^51^. Leaf chlorophyll content was a monitoring marker of overall plant health, particularly in the applied field, where plant health directly impacts productivity^52^. Several factors affected leaf quality, including chlorophyll distribution (decomposition and synthesis), nutrient content (distribution), and gene expression^53^. The content (Chl a + Chl b) and the ratio of chlorophyll (Chl a: Chl b) vary from species to species, but within a species, it should be specifically maintained for proper growth and long-time survival^54^. Maintenance of nutrient levels was essential for the proper functioning of photosynthetic machinery^55^. Any deficiency in pigment level will significantly impact plant growth, resulting in a decrease in net primary productivity^56^. The present study showed post-harvest retention of chlorophyll content in NS7 in comparison to FR0, while CO7 failed to do so. The magnitude of decrease in chlorophyll content in NS7 and CO7 was ~ 7% and ~ 69%, respectively, indicating better effectiveness of nanosilver solution in extending shelf life than distilled water used as a preservative. Quantitative measurement was supported by qualitative observation (morphological) showing the presence of yellowish patches in leaves preserved in distilled water, indicating senescence. Leaves preserved in nanosilver solution morphologically displayed retention of greenish texture almost similar to fresh leaves. The qualitative and quantitative observation was supported by expression analysis using NGS technology that was functionally annotated with *the Arabidopsis* database, the best annotated plant reference genome that carries detailed information about the functional identity of genes and gene products^57^. Annotated DEGs under photosynthetic processes (Light reaction: Photosystem I and Photosystem II; Dark reaction: Calvin cycle), development and assembly of chloroplast and chlorophyll are mostly up-regulated in NS7. Less expression of the photosynthetic and chloroplast-associated proteins in CO7 was the foremost cause for the activation of senescence-related processes. A decrease in water transport significantly promotes the decomposition of chlorophyll and accelerates leaf yellowing^54^. Our earlier study showed that during post-harvest preservation, blockage of xylem lumen by microbial proliferation and accumulation of secondary polymerized molecules interferes with the water-conducting pathway in CO7, promoting senescence and wilting^14^. Down-regulation of associated photosynthetic processes in CO7 was probably due to alterations in the structural and functional capability of the chloroplasts and associated proteins. The senescence-mediated cutback in photosynthesis was mainly due to the breakdown of protein functioning, pigment content, and membrane lipids degradation^58^. STRING round 2 qualified, topologically selected major chloroplast-associated up-regulated (NS7 vs. CO7) genes were AOR, PnsB3, PSBO2, CYP38, GLU2, EGY1, PSAA, PSAB, PSBA, PSBB, PSBD, and Lhca6. Of them PSAA, PSAB, PSBA, PSBB, PSBC, and PSBD were identified by KEGG to be directly associated with the photosynthetic process. PSAA and PSAB, which showed > 20-fold expression level, were encoded by the chloroplast genome and showed greater sequence homology^59^. PS I mediated electron transport system comprises PSAA and PSAB, remains associated with P700 in heterodimeric forms and with cascade components Ao (CHLa), A1 (phylloquinone), and Fx (4Fe-4S protein)^60^. It was reported that genomic expression of PSAA depends upon expression, synthesis, and thylakoid membrane integration of PSAB^61^. The current study showed a greater expression profile of PSAB than PSAA, supporting the above observation. It was observed that the synthesis and expression of one chloroplast-encoded protein promote the expression of another protein within the complex, i.e., co-expression of most photosynthesis-related genes occurs. For instance, PSBD (PS II as reaction center D2 protein) promotes the assembly of PSBA (subunit D1 of reaction center). Similarly, PSBB and PSBC encode core antenna subunits CP47 and CP43, promoting the binding of upstream oxygen-evolving enhancer (OEE) proteins^62^. This co-expressional mechanism was as per the earlier reports of Choquet and Vallon^63^ and Choquet and Wollman^64^. In contrast, the expressional profile of many photosynthetic proteins is correlated. For instance reduction in expression of PSBD proportionately reduces the expression of both PSBA and PSBB^65^. Similarly, the expression of PSBA and PSBB is also directly correlated^66^. In the present study, photosynthetic-related genes are observed to be differentially expressed. They are mostly up-regulated in NS7, and this is because most of the photosynthesis-associated genes are co-related. MCODE-mediated sub-networking analysis supports the above observation as it has generated two independent sub-networks (viz. chloroplast and photosynthesis and photosynthesis light reaction) associated with the photosynthetic process. HR-LCMS analysis of differentially expressed electrophoresis generated gel protein band of mulberry leaves preserved in nanosilver solution in our earlier report^67^ was in good agreement with the transcriptome profile, as the expression profile identified up-regulation of some common photosynthesis associated proteins (gene product). Carotenoids, which remain associated as a light-harvesting molecule with PSBB and PSBC63, were found to retain their content in NS7 until the last day of preservation. Carotenoids participate in non-photochemical quenching and thus act as a vital antioxidant for eliminating generated singlet oxygen^68^. Another important photosynthetic protein differentially expressed in NS7 was PSBO2 (an extrinsic subunit of PS II associated with water oxidizing complex). PSBO2 plays key role in stabilizing catalytic manganese cluster (Mn4Ca-cluster)^69^ and in the electron transport of PSII^70^. BiNGO identified the involvement of PSBO2 and Lhca6 in response to light stimulus (abiotic stress management). The defensive role of PSBO2 resides in its ability to dephosphorylate damaged D1 protein for degradation, thereby maintaining its turnover number^71^. Plant nano-bionics has identified the integration of biogenic nanoparticles into the chloroplast of the plant cell, thereby playing a potential role in extending the shelf life of crop plants^72^. The photosynthetic and chloroplast process maintenance was also carried out by an oxidoreductase, AOR representative of zinc-binding dehydrogenase protein family by detoxifying reactive carbonyl species formed during lipid peroxidation^73^.

Maintaining cellular redox homeostasis was the foremost prerequisite for extending shelf life at the post-harvest stage. In the post-harvest stage, the generation of oxidative stress can hardly ever be avoided. Thus, shelf life extension can only be achieved by mitigating generated ROS. An increase in the concentration of cellular oxygen molecules beyond the optimum level results in the generation of ROS either in the form of ^1^O_2_ (singlet oxygen) or the form of (superoxide), H_2_O_2_ (hydrogen peroxide), and ^·^OH (hydroxyl radical) by accepting electron^74^. Nucleus, chloroplasts, mitochondria, glyoxysomes, and peroxisomes are the key ROS generating sites, damaging various cellular components^75^. Carbohydrates, proteins, lipids, and nucleic acids, along with other cellular macro- and micro-molecules, are the target site of ROS action^76^. The current study showed a decrease in protein, and carbohydrate (total and reducing sugar) content in CO7 by ~ 71%, ~ 63%, and 64%, respectively, with respect to FR0, while NS7 can maintain the primary metabolite content almost like that of FR0. Whereas the content of stress markers viz*.* hydrogen peroxide, MDA, superoxide, and proline was significantly enhanced in CO7 by 11, 3, 30, and twofold, respectively, with respect to FR0. The content of stress markers has also increased in NS7, but the extent of enhancement was insignificant with respect to FR0. For nullifying cellular and sub-cellular damage caused by the imbalance of ROS, the tissue system requires an efficient antioxidative (enzymatic and non-enzymatic) repair system that can detoxify generated ROS^77^. Transcriptome analysis has identified a significant number of proteins involved in scavenging activity in NS7, including CSD1, LOX1, LOX6, and DOX1. CSD1 (superoxide dismutase) catalyzes the dismutation of superoxide generated by photosynthetic electron transport reactions to hydrogen peroxide^13,78^. KEGG identified the participation of LOX1 and LOX6 in linoleic acid metabolism, thereby participating in the jasmonic acid biosynthetic and metabolic process identified by BiNGO. LOX1, LOX6 (lipoxygenase), and DOX1 (α-dioxygenases) through the production of oxylipins involves in stress (biotic and abiotic) management^79^ by delaying senescence through controlled chloroplast destruction^80^. It has been reported that LOX1 mutant causes enhanced hydrogen peroxide and MDA accumulation, promoting cellular damage and causing senescence^81^. It has been stated that the activity of DOX1 increases in tomatoes and *Arabidopsis* leaves under oxidative stress generated due to bacterial proliferation^82^. In plants, DOX1 was reported to perform the tissue-protective role, and the degree of tissue damage was inversely proportional to the cellular level of α-dioxygenases^83^. The silver nanoparticle was reported to promote plant growth by preventing microbial and other pathogenic proliferations^84^. Implementation of nanosilver has identified more than 20 disease-resistance protein types. Among them significantly expressed defensive proteins were RPP8, PBS1, PAD4, and WAK1 in NS7. BiNGO identified the involvement of RPP8, PBS1, and PAD4 in innate immune response, which restricts pathogen growth through hypersensitive response^85^. PBS1 comes under the R-gene family and participates in effector-triggered immune response^86^. WAK1 was reported to be a transmembrane serine/threonine-protein kinase performing a diverse role in cell expansion, morphogenesis, and development^87^. WAK protein through cell wall integrity-sensing mechanism provides a defensive response against wounding, pathogen attack, and oxidative stress^88^. WAK1 and PAD4 were involved in MCODE-generated sub-network clustering involved in stress-associated defense response along with WRKY53, CRK10, CRK6, and AT3G28580. Another MCODE-generated defensive line identified was NAD-ME1, ALDH2B4, ALDH10A8, NADP-ME3, and PHS2. KEGG analysis recognized the involvement of NAD-ME1 and NADP-ME3 in the carbon fixation process of photosynthetic organisms as they participated in the oxidation of malic acid to pyruvic acid and carbondioxide^89^. Besides performing the metabolic role in the photosynthesis process, NAD-ME1, and NADP-ME3 are also reported to provide a defensive role by participating in the lignin biosynthesis process and maintaining cytoplasmic pH level^90^. ALDH2B4 and ALDH10A8 (aldehyde dehydrogenase) maintain aldehyde homeostasis within the cell and provides defense by scavenging toxic aldehydes^91^. In support of the present study, it was reported that ALDH genes were over-expressed for defense against abiotic stress^92^. ATR1 another defensive protein identified by MCODE and BiNGO involved in stress management by inducing hypersensitive response (HR), thereby providing resistance to bacterial pathogens^93^.

Dismantled chloroplast was associated with the degradation of internal macromolecules, causing a lack of nutrient accumulation and promoting senescence. Degradation of polymeric-molecules to monomeric form causes their transport through the phloem, altering sink–source relationship^94^. At the post-harvest stage, these molecules may get released through the cut end, accelerating senescence as observed in CO7. One such molecule was sucrose, whose internal concentration decreases upon senescence activation by the action of the enzyme invertase. Matsushita and Uritani^95^ reported an increased level of acid invertase in sweet potato tuber upon aging. The current study identified BFRUCT4 (acid beta-fructofuranosidase 4), which is acid invertase differentially expressed in CO7 (> 50 fold). BFRUCT4 probably causes continued mobilization and release of sucrose products through the cut end, decreasing internal sugar concentration and thereby decreasing shelf life. The involvement of BFRUCT4 in sucrose catabolism and transport, as reported by Aluru et al.^96^, which performed a vital role in sink metabolism, was in support of the present observation.

Leaf senescence is the summative response of multiple factorial operations working together in a stepwise manner. Senescence is a process of cell degeneration and death operated systematically under tight genetic control to enhance the plant's survival. The regulation and execution of senescence at the cellular and sub-cellular levels was controlled by the mode of action of different hormones, receptors, and transcription factors^97^. Most phyto-hormones viz*.* ethylene, abscisic acid, cytokinins, auxin, gibberellic acid, brassino steroids, jasmonic acid, and salicylic acid regulate both stress-induced and maturity dependent leaf senescence^98^. Ethylene, besides promoting senescence, involves in stress-related gene regulation and plant survival and growth response^99^. Cao et al.^100^ reported that CTR1 (constitutive triple response 1) mutant plants display higher longevity than wild-type plants due to continuous ethylene signaling. In the current study, CTR1 is down-regulated in NS7, indicating the ability of nanosilver solution to extend shelf life at the post-harvest stage. On the contrary, plants bearing over expression form of EIN4 become less tolerant to stress, promoting senescence as observed for CO7^98,101^. MCODE identified strong network interaction between RAN1, CTR1, and HMA5 in copper-mediated ethylene response. RAN1 and HMA5 was reported to work upstream of ETR1 (ethylene receptor 1), delivering copper to the receptor, and promoting ethylene binding^102^. In the ETR1 mutant, binding of copper ion was inhibited, and thus, ethylene binding was also ceased^103^. Similarly, in NS7, the down-regulation of ETR1 was due to the down-regulation of RAN1 and HMA5, inhibiting the process of senescence. ARF1 (auxin response factor 1) regulates senescence by regulating the expression of some senescence-associated genes^104^. It was reported that in the ARF1 mutant plant, expression of SAG12 (senescence-associated gene 12) gets suppressed; consequently, senescence gets suspended^105^. Down-regulation of ARF1 in NS7 was the probable reason for the long post-harvest shelf life.

Among different cellular and sub-cellular transporters, the expression profile of ABC class transporters gets uplifted during stress as they play a crucial role in detoxifying xenobiotics and transporting toxic substances^106^. A literature survey reveals that ABC transporters participate in abiotic stress response by excluding harmful cellular compounds, including heavy metals^107^. The current study identified three differentially expressed ABC transporters in CO7 viz*.* ABCC8, ABCG15, and ABCG33 forming MCODE generated separate sub-network. Class G-ABC transporters mainly regulate plant-pathogen defense by excluding microbial toxins from the plant body^108^. The accumulation of excessive toxic compounds inside CO7 causes enhanced expression of ABC class transporters to mitigate generated stress. The expression profile of ABCC8 gets enhanced during fruit ripening, and the process was related to chlorophyll degradation and anthocyanin accumulation in the fruit^109^. Similarly, enhanced expression of ABCC8 in CO7 was probably associated with the senescence-mediated leaf yellowing process. Another transporter differentially expressed in CO7 was CNGC4 (cyclic nucleotide-gated ion channel 4), a non-selective cation channel involved in defensive responses^110^. CNGC4, through enhancing cellular Ca^+^ level, participates in the expression of pathogenesis-related genes (PR-gene) and hypersensitive response^111^.

Phyto-immune system gets hyper-activated during stress and senescence management which leads to the identification of another MCODE-generated defensive line in CO7 viz. APS2, APS3, NIR1, WIN1, and PPC1. KEGG identified the involvement of APS2 (ATP-sulfurylase 2) and APS3 (ATP-sulfurylase 3) in metabolic pathways, including biosynthesis of amino acids and 2-oxocarboxylic acid metabolism. ATP-sulfurylase catalyzes the synthesis of adenosine-5-phosphosulfate, which gets incorporated into cysteine. Cysteine, through redox reactions, participates in varied abiotic stress management through the synthesis of antioxidant molecules, viz. glutathione, homo-glutathione, and phytochelatins^112^. Akbudak et al.^113^ stated that ATP sulfurylase activity was up-regulated under stress, probably for maintaining the pool of sulfur-containing amino acids, viz*.* methionine and cysteine, which will counter-balance generated ROS through the synthesis of antioxidant molecules. To mitigate generated toxic substances, CO7 has up-regulated the expression of APS2/3 inside the tissue system to protect against the rapid degradation of cell and cellular components. Similarly, NIR1 encodes nitrite reductase 1, one of the two enzymes along with glutamine-dependent asparagine synthase 1, in nitrogen assimilation during stress management^114^. PPC1 (Phosphoenolpyruvate carboxylase 1) was an indicator of dehydration stress, and its up-regulation in CO7 indicates excessive water loss due to interruption in the stomatal closure procedure^115^. In leaves, PPC1 generally participates in carbon and nitrogen metabolism^116^, but over expression was an indicator of osmotic stress condition^117^. Over expression of PPC1 in CO7 might be due to internal water deficit, which might occur due to the generated inability to conduct water because of xylem blockage, as reported in our earlier study^13^.

## Conclusion

Leaves preserved in nanosilver solution for seven days showed significant withholding of primary metabolites content, whereas leaves maintained in distilled water failed to retain and displayed senescence, as revealed by the yellowish texture of leaves and accumulation of stress markers. Transcriptome analysis identified significant retention of photosynthetic and chloroplast associated proteins in NS7. These proteins were mainly responsible for maintaining green texture and post-harvest shelf life through properly regulating photosynthetic machinery. Extension of shelf-life in NS7 was achieved probably through activation of the phyto-immune system involved in providing defense by scavenging toxic substances, including ROS. Probably inability to maintain cellular redox homeostasis, leakage of storage carbohydrates through the cut end, decrease in water transport, and failure to withhold nutrient levels caused rapid wilting associated senescence through the accumulation of ROS in CO7. However, enhanced expression of different transporters and immune modulators were observed in CO7, but their collective summation also failed to prolong the post-harvest shelf life.

## Materials and methods

### Plant sample and its preservation at post-harvest stage

For conducting the experimental work S1 cultivar of mulberry was selected. Fresh, healthy and disease free leaves of S1 cultivar was collected from Matigara Sericulture Complex, Siliguri, India (26°70′40″N and 88°35′37″ E). For collecting the leaf sample from Sericulture Farm permission was taken from Joint Director of Textile (Sericulture), North Zone, Siliguri through Office letter Memo No. 55/Dev-5 dated 10.04.2017. The experiment was carried out between April—October 2018–2019, and 2019–2020. Leaf samples were collected following the standard harvesting protocol setup Directorate of Textiles (Sericulture), Govt. of West Bengal, India during the daytime in wet gunny bags between 6:00–7:00 am to maintain the fresh texture of the leaves. The plant study complies with relevant institutional, national, and international guidelines and legislation. Within 30 min of collection, the leaves were transferred into the preservative solution. The leaves were preserved in 20 ml 6 ppm phytosynthesized silver nanoparticles^13^ and an equal volume of distilled water (pH 7) maintaining three replicated sets of each and each sets contains 15–20 leaves. Before inserting the petioles of the leaves within the preservative solution, a fine oblique cut of the petiole end was done to maintain the conducting column integrity^14^. The experimental setup was maintained at 260–270 lx, 40% humidity, and 25 °C for seven days. At the end of the preservation period, a portion of the preserved leaves was used for biochemical validation, and the remaining was used for transcriptome analysis.

### Biochemical validation of mulberry leaves post preservation

Validation of primary metabolite content was carried out concerning total chlorophyll, carotenoids, protein, total sugar, and reducing sugar content. Arnon method^118^ was followed for estimating total chlorophyll content. Preserved and fresh leaf samples were extracted twice with 20 ml of 80% (v/v) acetone, centrifuged for 5 min at 10,000×*g*, and maintained at 25 °C. UV–visible spectral readings (Systronics 2201) of the supernatants were taken at 663 and 645 nm for calculating chlorophyll content. For estimating carotenoid content, acetone supernatant used for chlorophyll estimation was taken, and spectral readings were measured at 470, 645, and 663 nm^119^.

The method of Lowry et al.^120^ was followed for estimating total protein content. Sample extraction was conducted using sodium phosphate buffer, centrifuged at 5000×*g* at − 10 °C for 5 min. The reaction mixture (blue color complex) containing supernatant (1 ml), alkaline copper solution (5 ml), and Folin-Ciocalteu reagent (0.5 ml) was measured spectrophotometrically at 660 nm after 30 min of incubation. A standard curve prepared from BSA (bovine serum albumin) was used for estimating the protein content.

Total soluble sugar content was measured following the process prescribed by Anthrone^121^. To 1 ml of extract, 4 ml Anthrone reagent was added and incubated for 10 min at 100 °C. The mixture was allowed to cool to room temperature, and absorbance was recorded at 620 nm. Total carbohydrate content was calculated by using the sucrose standard curve. DNSA method^122^ was followed to determine the amount of reducing sugar. To 1 ml 3,5-dinitrosalicylic acid (DNSA), sample extract was added and boiled for 5 min. In the colored product that was developed, to it 40% Rochelle salt solution was added, mixed well, and the mixture was brought to room temperature. Absorbance was taken at 510 nm, and reducing sugar content was calculated using a standard curve prepared from glucose.

### Estimation of stress marker content within mulberry leaves post preservation

Determination of stress accumulation within mulberry leaves post-preservation stage was evaluated with respect to hydrogen peroxide (H_2_O_2_), superoxide (O_2_^·−^), malondialdehyde (MDA), and proline content. The content of H_2_O_2_ was estimated at 390 nm using leaf supernatant (centrifuging at 10,000×*g* for 10 min at 4 °C) extracted in 1% (w/v) trichloroacetic acid (TCA) and reacted with 10 mM phosphate buffer (pH 7.0) and 1 M potassium iodide^123^.

Leaf tissue homogenised with 65 mM potassium phosphate buffer (pH 7.8) and centrifuging at 5000×*g* for 10 min was used for superoxide content estimation. The absorbance was recorded at 530 nm after amalgamating the supernatant with the reagent mixture containing 65 mM potassium phosphate buffer (pH 7.8), 10 mM hydroxylamine hydrochloride, 17 mM sulphanilamide, and 7 mM α-naphthylamine^124^.

MDA content was estimated for determining extent of lipid peroxidation by homogenizing the tissue in 2 ml 0.5% (w/v) TCA, centrifuged at 10,000×*g* for 10 min at 4 °C. The absorbance of the supernatant was recorded at 450, 532, and 600 nm after incubating the reaction mixture for 30 min at 95 °C containing 2 ml supernatant and 2 ml 0.67% thiobarbituric acid (TBA) and finally terminating the reaction using ice-cold water^125^.

Estimation of proline content was conducted according to the method prescribed by Bates et al.^126^. For estimation, leaf tissue was homogenized in 10 ml 3% sulfosalicylic acid, and the upper toluene layer was collected. Spectral reading of the upper toluene layer (1 ml) was taken at 520 nm after reacting with 2 ml acid ninhydrin, 2 ml glacial acetic acid, and 4 ml toluene.

### RNA extraction and high-throughput Illumina sequencing

Standard trizol extraction protocol was followed for RNA extraction from nanosilver and distilled water preserved (7 days) mulberry leaves. For extraction, 1 ml Trizol reagent (Aura biotechnologies, India) was mixed with 200 mg leaf tissue crushed in liquid nitrogen, vortex and incubated for 5 min at 25 °C. Following incubation, 0.2 ml chloroform (Himedia, India) per ml Trizol was added and agitated vigorously for 15 s and incubated at 25 °C for 3 min, followed by centrifuge at 14000×*g* for 15 min at 4 °C. To the upper aqueous phase, isopropyl alcohol was added for precipitating RNA, incubated for 5 min, followed by centrifugation at 12000×*g* for 10 min at 4 °C. The pellets were washed with 80% ethanol by vortex and centrifuged at 7000 × g for 5 min at 4 °C. The RNA pellet was dried and dissolved in 40 – 80 µl RNAase-free water and stored at 80 °C. RNAse-free agarose gel electrophoresis was performed to measure the integrity of isolated RNA^127^, and the quantity was measured using Bioanalyzer (Agilent Technologies, Santa Clara, CA). Total RNA from three replicates of NS7 and CO7 was used for library preparation and sequencing. Library preparation was conducted following the Illumina TrueSeq RNA library method as prescribed in TruSeq Stranded Total RNA Reference Guide (Illumina Technologies, San Diego, CA). Libraries of each sample (NS7 and CO7) were used for RNA sequencing using the Illumina HiSeq 4000 platform (Illumina Inc., CA, USA), which were exposed to automated cycles of paired-end-sequencing (2 × 100 bp).

### Pre-processing of RNA-Seq data set

Illumina raw reads were processed to remove adapter sequences using AdapterRemoval (version 2.2.0). Reads having an average quality score of < 20 were filtered out from the pair-end and reads. Trimming of rRNA reads was achieved using the Silva database (https://www.arb-silva.de/). The high-quality reads with a base quality score ≥ 30 were used for downstream analysis.

### de novo transcriptome assembly and differential gene expression analysis

Trinity (version 2.8.2) software package was used for de novo transcriptome assembly. Transcripts ≥ 200 bp was selected and used for further analysis. Quantification of transcript abundance was carried out using Kallisto (version 0.44.0)^128^ followed by differential gene expression analysis using the R package EdgeR (version 3.6)^129^. Prior to differential expression analysis, the raw counts obtained from Kallisto were normalized following Trimmed Mean of M-values (TMM) method^130^. The genes with log2FoldChange ≥ 2 and FDR < 0.05 were considered up-regulated, whereas the genes with log2FoldChange ≤ 2 and FDR < 0.05 considered down-regulated^131^. Significantly (FDR < 0.05), differentially expressed up-regulated and down-regulated genes were expressed in terms of MA, violin and volcano plot.

### Prediction of simple sequence repeats (SSRs)

Mulberry transcriptome was analyzed through the MIcroSAtellite identification tool (MISA, https://webblast.ipk-gatersleben.de/misa/) for detecting the presence of SSRs.

### Functional annotation of differentially expressed genes

Generalized annotation and validation of differentially expressed genes were performed with the NCBI plant NR database (http://www.ncbi.nlm.nih.gov/) and PMN (plant metabolic network) database (https://plantcyc.org/). While specific annotation with respect to *Arabidopsis* database was performed using Mercator (version 3.6) plaBi database^132^ (https://plabipd.de/portal/mercator-sequence-annotation), and revalidation was conducted with uniport database (https://www.uniprot.org/).

### Functional enrichment analysis of differentially expressed genes

Functional enrichment through protein–protein enrichment analysis was studied using STRING (version 11.0; https://string-db.org/). STRING generated biological process (BP), cellular component (CC), and molecular function (MF) were aligned with respect to percentile ranking of the obtained GO (Gene ontology) score for obtaining top rank processes. STRING-generated interaction data was passed through Cytoscape (version 3.7.1; https://cytoscape.org/) to obtain the topological gene score. Sub-networking analysis of highly interconnected nodes was performed using MCODE (version 2.0.0) statistical package considering node density cut from 0.1 and node score cut off to 0.2. Gene ontological study of topologically selected differentially expressed genes having ≤ 50% average percentile rank was performed using BiNGO plug-in (version 3.0.4) at Cytoscape platform. BiNGO process was used to identify GO terms through the application of the hypergeometric test, considering the *p* value cut off ≤ 0.05. KEGG analysis^133,134^ was done for determining the enriched pathways of screened up-regulated and down-regulated unigenes.

### Validation of differentially expressed genes by quantitative real-time PCR

For validating differentially expressed up-regulated and down-regulated genes, quantitative real-time PCR (qPCR) analysis was carried out using gene-specific primer sets. The standard trizol extraction protocol mentioned above was followed for RNA extraction. Extracted RNA was treated with DNase to avoid the possibility of DNA contamination. 10 μl of DNase-treated RNA was reacted with 5 × RT buffer (4 μl), 25 × dNTPs (0.8 μl), 10 × random primer (2 μl), reverse transcriptase (1 μl), and DEPC water (2.2 μl) for cDNA synthesis at ice-cold condition. The mixture was spun in the thermal cycler maintained at 25 °C for 10 min, 42 °C for 60 min, 85 °C for 5 min, and then held at 4 °C for ∞. Synthesized cDNA was stored at − 20 °C for RT-PCR analysis after confirmation by performing PCR with the housekeeping gene Actin 3.

The Primers designed for the gene expression studies were carried out with the Eurofin genomics PCR primer designing tool (https://eurofinsgenomics.eu/en/ecom/tools/pcr-primer-design/). Real-Time PCR relative quantification study was carried out in Applied Biosystems StepOne Real-Time PCR using the SYBR Green Chemistry (Sensifast SYBR HiRoxkit, Bioline, USA). The reaction mixture for qRT PCR contains cDNA (0.5 μl), 2 × SYBR Green Master Mix (5 μl), forward primer (10 μM, 0.5 μl), reverse primer (10 μM, 0.5 μl), and nuclease-free water (3.5 μl). qRT PCR program was operated as follows: pre-denaturation at 95 °C followed by 40 cycles of 15 s at 95 °C and 30 s at 60 °C, followed by the steps of dissociation curve generation (20 s at 95 °C, 60 s at 60 °C, and 15 s 95 °C). For accurate determination of gene expression values, raw fluorescence data (Ct values) generated by the real-time PCR instrument (Applied Biosystems) was exported to qBase plus software 13, which will scale raw data to an endogenous control gene (Actin). Relative expression of a target gene (TG) was done using the comparative Ct (ΔΔCt) method using StepOne Software (version 2.2.2) by applying the following equation: RQ = ${2}^{-\Delta \Delta \mathrm{CT}}= {2}^{\Delta \mathrm{CT }(\mathrm{target})}\times {2}^{\Delta \mathrm{CT }(\mathrm{reference})}$. Data was plotted graphically using OriginPro 2021software (9.8.0.200).

## Supplementary Information

Supplementary Figure 1.

Supplementary Figure 2.

Supplementary Figure 3.

Supplementary Figure 4.

Supplementary Figure 5.

Supplementary Figure 6.

Supplementary Figure 7.

Supplementary Figure 8.

Supplementary Figure 9.

Supplementary Figure 10.

Supplementary Figure 11.

Supplementary Figure 12.

Supplementary Figure 13.

Supplementary Figure 14.

Supplementary Legends.

Supplementary Table 1.

Supplementary Table 2.

Supplementary Table 3.

Supplementary Table 4.

Supplementary Table 5.

Supplementary Information 1.

Supplementary Information 2.

Supplementary Information 3.

Supplementary Information 4.

Supplementary Information 5.

Supplementary Information 6.

Supplementary Information 7.

Supplementary Information 8.

Supplementary Information 9.

Supplementary Information 10.

Supplementary Information 11.

Supplementary Information 12.

Supplementary Information 13.

Supplementary Information 14.

Supplementary Information 15.

Supplementary Information 16.

Supplementary Information 17.

Supplementary Information 18.

Supplementary Information 19.

Supplementary Information 20.

Supplementary Information 21.

## Data Availability

The datasets of clean raw read sequences from each tissue sample (NS7 and CO7) were deposited in the National Centre for Biotechnology Information (NCBI) which can be accessed in the form of sequenced read archive (SRA) under accession number SRR9665629 (https://www.ncbi.nlm.nih.gov/sra/?term=SRR9665629) and SRR9665368 (https://www.ncbi.nlm.nih.gov/sra/?term=SRR9665368) for NS7 and CO7 respectively. Both the SRA were registered under the same bio-project and bio-sample accession number PRJNA553319 and SAMN12234591respectively.
