# Supplementary material for: Transcriptome analysis of mulberry (Morus alba L.) leaves to identify differentially expressed genes associated with post-harvest shelf-life elongation
Source: Sci Rep. 2022 Oct 28;12:18195. doi: 10.1038/s41598-022-21828-7 (PMC9616847; doi:10.1038/s41598-022-21828-7)
Supplement: Supplementary file 15 — Supplementary Legends. [file 41598_2022_21828_MOESM15_ESM.docx]

**Supplementary Fig. 1** Score plot analysis of principal components demonstrating preservative potential of distilled water (CO7) and nanosilver solution (NS7) in preserving mulberry leaves at post harvest stage with respect to fresh leaves (FR0).

**Supplementary Fig. 2** Overview of transcriptome assembly through sequence length distribution between assembled transcripts and unigenes.

**Supplementary Fig. 3** Comparative histogram of GC range distribution between assembled transcripts and unigenes.

**Supplementary Fig. 4** The volcano maps showing differentially expressed up-regulated and down-regulated (**A**) isoforms and (**B**) unigenes. Green dots represent up-regulated genes while red dots represent down-regulated genes (p < 0.05).

**Supplementary Fig. 5** STRING analysis of differentially expressed up-regulated genes obtained after annotation with Arabidopsis database using Mercator and Uniport. STRING network contains 441 node connected by 4651 edges having PPI enrichment of p< 1.0e-16.

**Supplementary Fig. 6** STRING analysis of differentially expressed down-regulated genes obtained after annotation with Arabidopsis database using Mercator and Uniport. STRING network contains 572 node connected by 4541 edges having PPI enrichment of p< 1.0e-16.

**Supplementary Fig. 7** Gene Ontology classification of top differentially expressed up-regulated genes with respect to biological process (BP1), cellular component (CC1) and molecular function (MF1) obtained from STRING analysis.

**Supplementary Fig. 8** Enzyme Class classification of differentially expressed up-regulated genes

**Supplementary Fig. 9** Gene Ontology classification of top differentially expressed down-regulated genes with respect to biological process (BP1), cellular component (CC1) and molecular function (MF1) obtained from STRING analysis

**Supplementary Fig. 10** Enzyme Class classification of differentially expressed down-regulated genes

**Supplementary Fig. 11** STRING second round analysis of selected up-regulated unigenes those are found to be significantly enriched from round one GO analysis. STRING network contains 71 node connected by 298 edges having PPI enrichment of p< 1.0e-16.

**Supplementary Fig. 12** STRING second round analysis of selected down-regulated unigenes those are found to be significantly enriched from round one GO analysis. STRING network contains 82 node connected by 216 edges having PPI enrichment of p< 1.0e-16.

**Supplementary Fig. 13** Expression profile of topologically selected top up-regulated genes.

**Supplementary Fig. 14** Expression profile of topologically selected top down-regulated genes.
