# Supplementary material for: Transcriptome analysis of mulberry (Morus alba L.) leaves to identify differentially expressed genes associated with post-harvest shelf-life elongation
Source: Sci Rep. 2022 Oct 28;12:18195. doi: 10.1038/s41598-022-21828-7 (PMC9616847; doi:10.1038/s41598-022-21828-7)
Supplement: Supplementary file 16 — Supplementary Table 1. [file 41598_2022_21828_MOESM16_ESM.docx]

**Supplementary Table 1** Summary of assembled transcript

| **Description** | **No. of Assembled Transcripts** | **No. of unigenes** |
| --- | --- | --- |
| **Number of assembled transcripts** | 157982 | 81952 |
| **Longest transcript length (bp)** | 24890 | 79047 |
| **Mean GC % of transcripts** | 41.02 | 39.97 |
