# Supplementary material for: Transcriptome analysis of mulberry (Morus alba L.) leaves to identify differentially expressed genes associated with post-harvest shelf-life elongation
Source: Sci Rep. 2022 Oct 28;12:18195. doi: 10.1038/s41598-022-21828-7 (PMC9616847; doi:10.1038/s41598-022-21828-7)
Supplement: Supplementary file 17 — Supplementary Table 2. [file 41598_2022_21828_MOESM17_ESM.docx]

**Supplementary Table 2** Contigs summary based on all Trinity transcript and unigenes

| **Particulars** | **Trinity transcript (Isoform)** | **Unigenes** |
| --- | --- | --- |
| Contig N10 | 4939 | 4325 |
| Contig N20 | 3731 | 3178 |
| Contig N30 | 3031 | 2459 |
| Contig N40 | 2507 | 1896 |
| Contig N50 | 2054 | 1374 |
| Median contig length | 585 | 365 |
| Average contig | 1116.27 | 730.6 |
| Total assembled bases | 176350155 | 59874339 |
