# Supplementary material for: Transcriptome analysis of mulberry (Morus alba L.) leaves to identify differentially expressed genes associated with post-harvest shelf-life elongation
Source: Sci Rep. 2022 Oct 28;12:18195. doi: 10.1038/s41598-022-21828-7 (PMC9616847; doi:10.1038/s41598-022-21828-7)
Supplement: Supplementary file 18 — Supplementary Table 3. [file 41598_2022_21828_MOESM18_ESM.docx]

**Supplementary Table 3** Category wise allocation of predicted SSRs

| **Types of Repeats** | **p1** | **p2** | **p3** | **p4** | **p5** | **p6** | **c** | **c*** |
| --- | --- | --- | --- | --- | --- | --- | --- | --- |
| Total number of SSRs predicted | 38557 | 18611 | 8508 | 989 | 246 | 107 | 10588 | 479 |

(p1, p2, p3, p4, p5, p6 encodes SSR based on monomer, dimer, trimer, tetramer, pentamer, hexamer respectively. c and c* encodes SSRs without and with an interruption between two motifs respectively.)
