# Supplementary material for: Transcriptome analysis of mulberry (Morus alba L.) leaves to identify differentially expressed genes associated with post-harvest shelf-life elongation
Source: Sci Rep. 2022 Oct 28;12:18195. doi: 10.1038/s41598-022-21828-7 (PMC9616847; doi:10.1038/s41598-022-21828-7)
Supplement: Supplementary file 19 — Supplementary Table 4. [file 41598_2022_21828_MOESM19_ESM.docx]

**Supplementary Table 4** Summary of differentially expressed isoforms and unigenes

| **NS7 vs CO7** | **Up Regulated** | **Down Regulated** |
| --- | --- | --- |
| Isoforms | 2112 | 2806 |
| Unigenes | 1587 | 1826 |
