# Supplementary material for: Transcriptome analysis of mulberry (Morus alba L.) leaves to identify differentially expressed genes associated with post-harvest shelf-life elongation
Source: Sci Rep. 2022 Oct 28;12:18195. doi: 10.1038/s41598-022-21828-7 (PMC9616847; doi:10.1038/s41598-022-21828-7)
Supplement: Supplementary file 20 — Supplementary Table 5. [file 41598_2022_21828_MOESM20_ESM.docx]

**Supplementary Table 5** Primer sequences for RT-PCR amplification

| **Genes** | **Primer type** | **Sequence** | **Tm** |
| --- | --- | --- | --- |
| PSBB | Fw | TCCTTTTTCAAACCCTGCTG | 55.25 |
|  | Rv | TCTTTAAATTCTGTGGGTGGC | 55.92 |
| CSD1 | Fw | ACCAAAAGCCAGCAGAAAAC | 60.25 |
|  | Rv | GAGGAGAGTGAGAGAAGAAGAG | 61.12 |
| PSBO2 | Fw | ACACTCTCAAACACACCAATC | 55.92 |
|  | Rv | AGTTTTTAGTCCCGTCCTACC | 57.87 |
| AOR | Fw | CCAACCTCTACAACCTCTCC | 59.35 |
|  | Rv | TCGAAGCAACGAGTATAAATCC | 56.53 |
| RAN1 | Fw | ATGCATTCGGAACGAGAGCC | 59.35 |
|  | Rv | GCGAATAGCGCCTTTTTCTCAG | 60.25 |
| ABCC8 | Fw | TGTCTGTGTTCTGTCTTTGTTC | 56.53 |
|  | Rv | TCCGTATTCTCATCACCAGTC | 57.87 |
| BFRUCT4 | Fw | ACCCTCTCTCTCTCTCTCTC | 59.35 |
|  | Rv | TGTGCTTCATCAATGTCGTC | 55.25 |
| CNGC4 | Fw | ACGCCTTCGTCATACTTCC | 56.67 |
|  | Rv | CAAGAACATACCAACAACCCC | 57.87 |
